# Dynamic modeling of soft continuum manipulators using lie group variational integration

**DOI:** 10.1371/journal.pone.0236121

**Published:** 2020-07-22

**Authors:** Abbas Tariverdi, Venkatasubramanian Kalpathy Venkiteswaran, Ørjan Grøttem Martinsen, Ole Jacob Elle, Jim Tørresen, Sarthak Misra

**Affiliations:** 1 Department of Physics, University of Oslo, Oslo, Norway; 2 Department of Biomechanical Engineering, University of Twente, Enschede, The Netherlands; 3 Department of Clinical and Biomedical Engineering, Oslo University Hospital, Oslo, Norway; 4 The Intervention Centre, Oslo University Hospital, Oslo, Norway; 5 Department of Informatics, University of Oslo, Oslo, Norway; 6 Department of Biomedical Engineering, University of Groningen and University Medical Centre Groningen, Groningen, The Netherlands; Beijing University of Posts and Telecommunications, CHINA

## Abstract

This paper presents the derivation and experimental validation of algorithms for modeling and estimation of soft continuum manipulators using Lie group variational integration. Existing approaches are generally limited to static and quasi-static analyses, and are not sufficiently validated for dynamic motion. However, in several applications, models need to consider the dynamical behavior of the continuum manipulators. The proposed modeling and estimation formulation is obtained from a discrete variational principle, and therefore grants outstanding conservation properties to the continuum mechanical model. The main contribution of this article is the experimental validation of the dynamic model of soft continuum manipulators, including external torques and forces (e.g., generated by magnetic fields, friction, and the gravity), by carrying out different experiments with metal rods and polymer-based soft rods. To consider dissipative forces in the validation process, distributed estimation filters are proposed. The experimental and numerical tests also illustrate the algorithm’s performance on a magnetically-actuated soft continuum manipulator. The model demonstrates good agreement with dynamic experiments in estimating the tip position of a Polydimethylsiloxane (PDMS) rod. The experimental results show an average absolute error and maximum error in tip position estimation of 0.13 mm and 0.58 mm, respectively, for a manipulator length of 60.55 mm.

## 1 Introduction

Reachability, high level of dexterity, and large elastic deformability are the primary driving factors behind the growth of research in the design, modeling, and control of continuum manipulators. Flexible continuum manipulators have recently generated interest in several fields [1–3], especially in minimally invasive surgical robotics and interventional medicine, such as catheter-based endovascular intervention [4, 5] and cardiac surgeries [6, 7]. In contrast to conventional rigid link manipulators, soft manipulators are able to reshape their configurations to allow for redundancies in path planning, and are capable of precise and delicate manipulation of objects in complex and varying environments.

There are numerous candidate actuation mechanisms for continuum manipulators such as tendon-drives and concentric tubes [8–11]. Compared to other actuation mechanisms, magnetic actuation benefits from high dexterity, versatility, and wireless actuation [12–15]. By applying remote magnetic torques on permanent magnets or coils which are embedded inside the body of a manipulator and/or at its tip, one can control the motion and configuration of the manipulator.

This paper aims to develop a computational model for analyzing the dynamics of soft continuum manipulators, which is one of the key challenges in soft robotics. In many tasks, dynamic models of manipulators are essential for control, trajectory planning, and optimal design purposes, especially in Minimally Invasive Surgeries (MIS) for operation in unknown and unstructured environments such as inside the human body. Due to elastic characteristics and geometric nonlinearities (i.e., bending, torsion, shear, elongation, including large deformation) of continuum manipulators, their dynamics have highly nonlinear behavior and are expressed as partial differential equations. Some recent modeling approaches of soft continuum manipulators/robots, which have been employed in the surgical robotics field, are summarized in Table 1.

**Table 1:** References on dynamics/static analysis of soft continuum manipulators in surgical robotics field.

References	Modeling Approach and its Properties	Robot type/ Application
[16]	Static analysis: Cosserat rod model. 3D elesticity	Surgical suture/ strands
[17]	Beam mechanics based on elastic energy	Concentric tubes/ General MIS
[18]	Static analysis based on screw theory and a virtual-work model	Multiple parallel backbones/ General MIS
[10, 19]	Linear elasticity theory	Single/Redundant tendons
[20]	3D Static analysis with loads: Cosserat rod model	General purpose CRs
[21]	Beam mechanics based on elastic energy (includes both bending and torsion)	Concentric tubes/ General MIS
[22]	Bernoulli–Euler elastica theory: statics, 2D	Multibackbone
[23]	Static analysis based on a virtual-work model	Serial Segments/ General surgical end-effectors
[24, 25]	Static analysis: Cosserat rod theory	Concentric tubes with and without external loads
[26]	Static analysis: Cosserat rod theory	Magnetic Catheter/ General purposes
[27]	Loaded static analysis: Cosserat rod theory	General MIS
[28]	Dynamic analysis: Cosserat rod model. 3D elesticity	Guidewire/ Interventional Radiology procedures
[29]	FEM: large deformation and inflation	Simulations on general medical robots
[30]	Lumped-parameter model	Multiple parallel shafts/ general Magnetic resonance imaging (MRI)-compatible medical manipulators
[31]	Pseudo-rigid-body model	Multiple parallel shafts/ cardiac robotic catheter
[32]	2D static analysis: rigid-link modeling	Planar tendon-driven continuum manipulator/ general medical robots
[33]	Static analysis: *α* Lie group formulation	Planar continuum: simulations and bechmark analysis/ intravascular shaping operations
[34]	3D static analysis: pseudo rigid body model	Magnetic catheter/ General surgical catheters

Soft continuum manipulators are analogous to specific Cosserat continuums. Therefore, Lie group synchronous variational integrators [35, 36], a novel time and space integration scheme, is employed in this paper to model geometrically exact beams based on the Simo beam model [37] and Hamiltonian formulation. The core idea of this algorithm is to obtain the dynamic behavior of the system while conserving the invariants (energy, momentum maps) of the system, especially for long-time simulations. The distinguishing characteristic of variational integrators is that they define the equations of motion based on the discretized variational principle of the system. Combining the integrators with Lie-group/algebraic techniques enables the algorithm to respect not only the structure of the dynamics and its properties but also preserve the structure of the configuration space. The advantages of employing the Lie group variational integration method compared to other modeling strategies is that the proposed solver is applicable to exact nonlinear dynamic models of continuum manipulators subject to large deformations. The algorithm preserves the symplectic structure, i.e., the invariants of mechanical systems. Also, it allows the usage of different time steps at different points in a given finite element for the geometry of soft manipulators. These properties are investigated in previous work (e.g., [35, 38, 39]), while the main focus of this paper is the experimental validation of the method on magnetically-actuated soft continuum manipulators.

Investigation of previous work in modeling of the continuum manipulators suggests that existing literature focuses primarily on static or quasi-static approaches, or does not provide sufficient experimental validation in realistic application scenarios. By contrast, the main contribution of this article compared with the existing work in literature is the validation of an accurate dynamic model of a soft continuum manipulator, considering spatial motion. Also, it should be noted that the model accounts for the geometric nonlinearities (e.g., large deformation) and respects conservation of dynamical properties of the system (e.g., energy and momentum maps conservation), and structures of configuration space simultaneously. Besides, it should be pointed out that three-dimensional internal and external dissipation forces act on the continuum manipulator and hence affect the dynamics. Therefore, it is necessary to consider these friction/ dissipation forces in the validation process. To this end, distributed prediction filters have been proposed.

In summary, this article’s contributions can be stated as follows.

- Existing studies on the modeling of continuum manipulators primarily consider static or quasi-static approaches. However, in numerous applications, the fully spatial dynamics of manipulators need to be considered for accurate control and design purposes. The primary contribution of this article is the derivation and experimental validation of a dynamic model for forced continuum manipulators with soft materials undergoing spatial deformation. The model accounts for the nonlinearities of the continuum manipulator; loading resulted from magnetic fields, the gravity, and internal and external dissipation forces generated by friction.
- Due to the difficulty in obtaining knowledge about the internal and external dissipation forces, distributed estimation filters have been designed to take these forces into account and capture their behavior.

The rest of the paper is organized as follows. In Section 2, mathematical preliminaries, including the system description and notation, are discussed. Next, Section 3 addresses the algorithm and numerical results. The experimental framework and implementation results are described in Section 4, demonstrating the effectiveness of the theoretical formulation. In addition, Section 5 provides a discussion on the implementation of the modeling algorithm. Finally, Section 6 summarizes the results of this work and draws conclusions and posits directions for future work.

## 2 Continuum manipulator dynamics

This section is devoted to describing kinematics and full three-dimensional dynamics for continuum manipulators undergoing large deflections (for detailed explanations, refer to the reference [35]). We review the static description of a continuum manipulator in three-dimensional space $R^{3}$ toward deriving the dynamic equations of motion of geometrically exact continuum manipulator by applying Hamilton’s principle to the Lagrangian of the system.

### 2.1 Kinematics

The manifold of configuration space of a continuum manipulator considering Boundary Conditions (BCs) is defined as

$$\begin{array}{c} Q=\{\left(O,P\right)\in C^{\infty }\left(\cdot \right):\left[0,L\right]\to SO\left(3\right)\times R^{3}|BCs\;are\;satisfied\} \end{array}$$

in which *L* is the length of the undeformed continuum manipulator, $P:\left[0,L\right]\to R^{3}$ maps the line of continuum manipulator’s centroids (i.e. center of mass) to Euclidean space $R^{3}$ and the orthogonal transformation O:[0,L]→SO(3) determines the orientation of moving cross-sections at points P(s) in the terms of a fixed basis $\left\{E_{1}\left(s\right),E_{2}\left(s\right),E_{3}\left(s\right)\right\}$. Therefore, the orientation of each cross-section which is denoted by directors or moving basis $\left\{D_{1}\left(s\right),D_{2}\left(s\right),D_{3}\left(s\right)\right\}$ can be written as

$$\begin{array}{c} D_{i}\left(s\right)=O\left(s\right)E_{i},\;i=1,2,3. \end{array}$$

**Fig 1:**
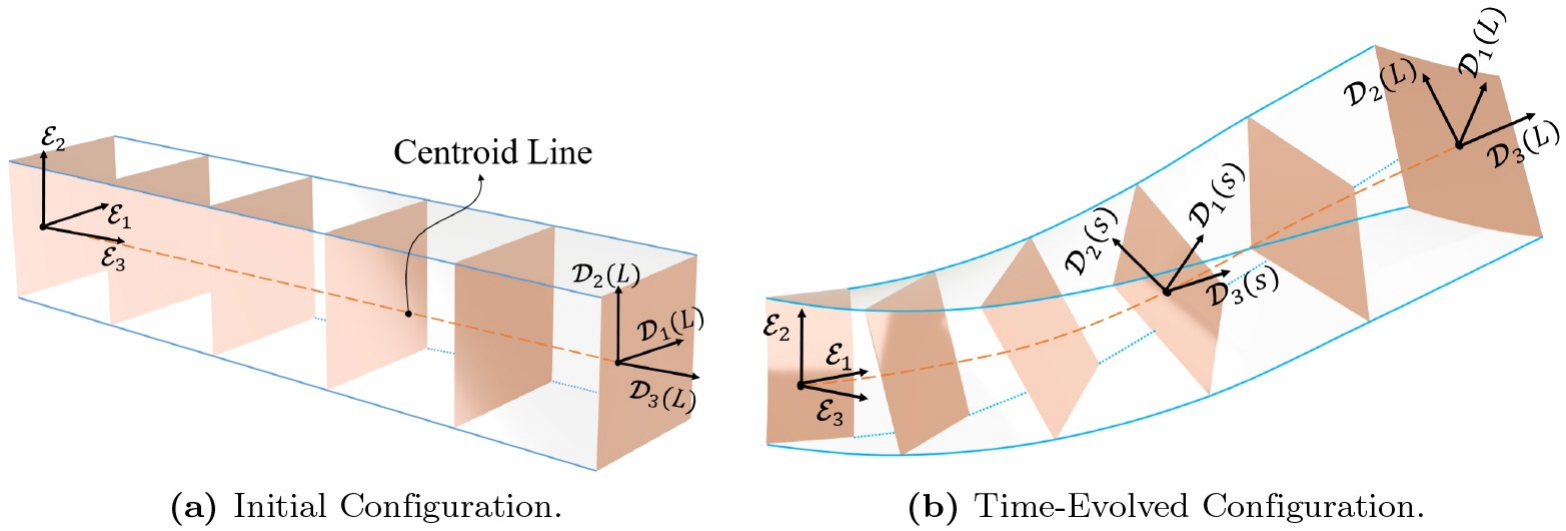
Initial and time-evolved configurations of the continuum manipulator. The highlighted frames depict cross-sections at discretization points. Fixed bases or material frame $\left\{E_{1},E_{2},E_{3}\right\}$ are also shown at the fixed end of the manipulators. Also, moving bases $\left\{D_{1},D_{2},D_{3}\right\}$ are attached to the cross section at the centroid *s* and the tip of manipulators.

Fig 1 shows initial and a time-evolved configuration of the continuum manipulator with the free right tip and clamped left end, i.e, BCs: $O\left(0\right)=I_{3}$, $\frac{\partial P(0)}{\partial s}=E_{3}$. The BCs imply that the clamped cross section is orthogonal to the plane defined by $E_{1}$ and $E_{2}$. In addition, a curve q(s,t)=(O(s,t),P(s,t))∈Q characterizes a time-evolved configuration space of the continuum manipulator. The family of tangent vectors to the curve *q*(*t*) is defined as

$$\begin{array}{c} \dot{q}\left(s,t\right)=\frac{\text{d}q(s,t)}{\text{d}t}=(\dot{O}\left(s,t\right),\dot{P}\left(s,t\right))\in T_{q}Q, \end{array}$$

which characterize tangent bundle $T_{q}Q$ to Q at the manifold *q*(*s*, *t*).

### 2.2 Lagrangian and equation of motion

To derive the equations of motion, we first need to introduce the Lagrangian $L:T_{q}Q$ of the system which can be written as

$$\begin{array}{c} L\left(O,P,\dot{O},\dot{P}\right)=\underset{\text{Kinetic}\;\text{energy}}{\underset{︸}{\frac{1}{2}\int_{0}^{L}(M\|\dot{P}{\|}^{2}+\omega^{T}J\omega )\text{d}s}}\\ \;-\underset{\text{Elastic}\;\text{energy}}{\underset{︸}{\frac{1}{2}\int_{0}^{L}({\left(\Gamma -E_{3}\right)}^{T}C_{1}\left(\Gamma -E_{3}\right)+\Omega^{T}C_{2}\Omega )\text{d}s}}-\underset{\text{Conservative}\;\text{potential}\;\text{energy}}{\underset{︸}{\int_{0}^{L}F^{c}\cdot P\text{d}s}} \end{array}$$ (1)

where the matrices *C*_1_ and *C*_2_ are defined as *C*_1_ ≔ diag(*GA GA EA*) and *C*_2_ ≔ diag(*EI*_1_ *EI*_2_ *GJ*^*p*^). For brevity, other parameters are defined in Table 2.

**Table 2:** Definition of parameters in Lagrangian (1) as described in [35].

*M* = *ρ*_0_ × *A*	*ρ*_0_ and *A* are the body constant mass density and cross section’s area.
ω(s,t)∈so(3)	the body angular velocity
$J=-\rho_{0}\int_{A}{\hat{\left(xE_{1}+yE_{2}\right)}}^{2}dxdy$	inertia matrix in the fixed frame
$\left(\Omega \left(s,t\right),\Gamma \left(s,t\right)\right)=\left(O^{-1}\frac{\partial O}{\partial s},O^{-1}\frac{\partial P}{\partial s}\right)$	deformation gradients as viewed at the time *t* by an observer that is located at the position *s*
*E*, *G* = *E*/(2(1 + *ν*)), *ν*, *I*_1_, *I*_2_, and *J*^*p*^	Young’s modulus, shear modulus, Poisson’s ratio, principal moments of inertia of the cross-section, and polar moment of inertia, respectively.

where each cross section is given by a compact set A={(x,y)|x,y∈R}, Lie algebra so(3) is associated with the Lie group *SO*(3), and Hat map/ operator ^:ℝ3→so(3) which is a one-to-one invertible map, i.e., an isomorphism, is defined as

$$v=[\begin{array}{c} v_{1}\\ v_{2}\\ v_{3} \end{array}]\to \hat{v}=[\begin{array}{ccc} 0 & -v_{3} & v_{2}\\ v_{3} & 0 & -v_{1}\\ -v_{2} & v_{1} & 0 \end{array}].$$ (2)

The Euler-Lagrange equations are obtained by applying by the Lagrange-d’Alembert principle to the action functional H associated to L, namely

$$\begin{array}{c} Y\left(O,P\right)=\int_{t_{o}}^{t_{f}}(L\left(O,P,\dot{O},\dot{P}\right)+F^{nc}\left(O,P,\dot{O},\dot{P}\right))\text{d}t \end{array}$$

By employing the Lagrange-d’Alembert principle, one computes

$$\begin{array}{c} \delta Y=\int_{t_{o}}^{t_{f}}(\int_{0}^{L}(M{\dot{P}}^{T}\left(\delta \dot{P}\right)+\omega^{T}J\delta \omega )\text{d}s\\ \;-\int_{0}^{L}({\left(\Gamma -E_{3}\right)}^{T}C_{1}\delta \Gamma +\Omega^{T}C_{2}\delta \Omega )\text{d}s\\ -\int_{0}^{L}F^{c}\delta P\;\text{d}s-F^{nc}\cdot \delta q\left(s,t\right))\text{d}t \end{array}$$

The terms *δω*, *δ*Ω, and *δ*Γ are defined ([38]) as follows:

$$\begin{array}{c} \begin{array}{cc} \delta \omega & =\omega \times \eta +\frac{\text{d}}{\text{d}t}\eta \\ \delta \Omega & =\frac{\partial }{\partial s}\eta +\Omega \times \eta \\ \delta \Gamma & =O^{T}\delta (\frac{\partial }{\partial s}P)+\Gamma \times \eta \end{array} \end{array}$$ (3)

where $\delta O=O\hat{\eta }$.

Taking into account the expressions for *δω*, *δ*Ω, and *δ*Γ in Eq (3) and using integration by parts in space and time, we obtain Euler-Lagrange equations with non-conservative force $F^{nc}\left(O,P,\dot{O},\dot{P}\right):T_{q}Q\to T_{q}^{*}Q$ as

$$\begin{array}{c} \begin{array}{cc} J\dot{\omega }-J\omega \times \omega -\Gamma \times C_{1}\left(\Gamma -E_{3}\right)-\Omega \times C_{2}\Omega -C_{2}\frac{\partial \Omega }{\partial s} & =O^{-1}N\\ M\ddot{P}-\frac{\partial OC_{1}\left(\Gamma -E_{3}\right)}{\partial s}+F^{c} & =F \end{array} \end{array}$$ (4)

in which we could consider 6 × 1 representations of general non-conservative force vector $F^{nc}=\left[\frac{N}{F}\right]$ where F and N are force and moment vectors in $R^{3}$, respectively. Also, $T_{q}^{*}Q$ denotes the cotangent bundle of Q. For simplicity, one may think of the cotangent boundle as the space of positions and momenta. For the exact definitions, refer to [40] or [41].

## 3 Lie group variational integrators for the forced continuum manipulator

In this section, the focus is on analyzing a Lie group variational integrator for continuum manipulators with conservative (e.g., the gravity) and non-conservative forces (e.g., friction and loads inserted by actuators). In the following subsection, the discretized version of the forced Euler-Lagrange Eq (4) is given (for further details and stability analysis, see [35]), and afterward, the estimation process is discussed.

### 3.1 Modeling

**Fig 2:**
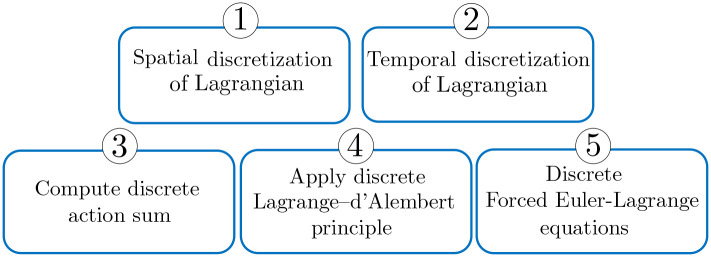
Steps 1 through 5 toward deriving continuum manipulator discrete dynamics.

This section is devoted to introducing a Lie group variational integration scheme for continuum manipulators with external loading. First, one needs to consider the spatial discretization of Lagrangian introduced in the previous section. Afterward, discrete Lagrange-d’Alembert equations need to be expressed on Lie group *SE*(3). These equations are employed to propose a model-based distributed estimation scheme. Fig 2 depicts the modeling procedure in this section.

Here notations of the paper are provided. Additionally, concepts and definitions on Lie groups and Lie algebra are presented in Appendix 6.

**Notations**: The undeformed continuum manipulator’s length [0, *L*] is spatially discretized into *N* subsets $I_{i}=\left[s_{a_{i}},s_{a_{i+1}}\right]$ of length $l_{I_{i}}=s_{a_{i+1}}-s_{a_{i}}$. For an element $I_{i}$, *a*_*i*_ and *a*_*i*+1_ denote its left and right nodes. The configuration of the continuum manipulator at the node *a*_*i*_ is given by $O_{a_{i}}:=O\left(s_{a_{i}}\right)$ and $p_{a_{i}}:=P\left(s_{a_{i}}\right)$. Also, $\omega_{a_{i}}$ denotes the angular velocity of a node *a*_*i*_. Given a node *a*_*i*_, the discrete time evolution of this node is given by the discrete curve $\left(O_{a_{i}}^{j},p_{a_{i}}^{j}\right)\in SE\left(3\right)=SO\left(3\right)\times R^{3},\;j=0,\cdots ,V$ and is based on the discrete Euler-Lagrange equations on Lie group *SE*(3), The discrete variables $F_{a_{i}}^{j}$ associated with the node *a*_*i*_ are defined as $F_{a_{i}}^{j}={\left(O_{a_{i}}^{j}\right)}^{T}O_{a_{i}}^{j+1}$. We denote the fixed time step by Δ*t* = *t*_*j*_ − *t*_*j*+1_, *j* = 0, ⋯, *V*. In time discretization of the continuum manipulator, we have $\Delta p_{a_{i}}^{j}:=p_{a_{i}}^{j+1}-p_{a_{i}}^{j}$.

By identifying the configuration space Q of the continuum manipulator with the infinite dimensional Lie group $G=C^{\infty }(\left[0,L\right],SO\left(3\right)\times R^{3}$, we consider the trivialized Lagrangian L:G×g→R, where g is a Lie algebra associated with the Lie group *G*. A spatial discretization of the trivialized Lagrangian for an element $I_{i}$ and the total system are computed as follows, respectfully. It should be noted that the evaluation of Lagrangian at midpoints of nodes is employed. Other evaluations of the Lagrangian depending on a different number or combinations of nodes are possible (see [38]).

For an element $I_{i}$:

$$\begin{array}{c} L_{I_{i}}=\frac{l_{I_{i}}}{4}M(\|p_{a_{i}}{\|}^{2}+{\left\|p_{a_{i+1}}\right\|}^{2})+\frac{l_{I_{i}}}{4}(\omega_{a_{i}}^{T}J\omega_{a_{i}}+\omega_{a_{i+1}}^{T}J\omega_{a_{i+1}})-V_{I_{i}} \end{array}$$ (5)

where $V_{I_{i}}$ is conservative potential energy of an element $I_{i}$ due to the gravity and elasticity and given by

$$\begin{array}{c} V_{I_{i}}=\frac{l_{I_{i}}}{4}[(O_{a_{i}}^{T}\frac{\Delta p_{a}}{l_{I_{i}}}-E_{3}{)}^{T}C_{1}(O_{a_{i}}^{T}\frac{\Delta p_{a}}{l_{I_{i}}}-E_{3})+(O_{a_{i+1}}^{T}\frac{\Delta p_{a}}{l_{I_{i}}}-E_{3}{)}^{T}C_{1}(O_{a_{i+1}}^{T}\frac{\Delta p_{a}}{l_{I_{i}}}-E_{3})] \end{array}$$ (6)

For the whole continuum manipulator:

$$\begin{array}{c} L=\sum_{i=1}^{N}(\frac{l_{I_{i}}}{2}M{\left\|p_{a_{i}}\right\|}^{2}+\frac{l_{I_{i}}}{2}\omega_{a_{i}}J\omega_{a_{i}})+\sum_{i=0,N+1}(\frac{l_{I_{i}}}{4}M{\left\|p_{a_{i}}\right\|}^{2}+\frac{l_{I_{i}}}{4}\omega_{a_{i}}J\omega_{a_{i}})\\ -\sum_{i=0}^{N+1}V_{I_{i}} \end{array}$$

The temporal discretized Lagrangian $L_{I_{i}}^{j}$ approximates the Lagrangian $L_{I_{i}}$ in Eq (5) during the time step Δ*t* is therefore

$$\begin{array}{c} L_{I_{i}}^{j}=\sum_{a=a_{i},a_{i+1}}(\frac{l_{I_{i}}}{4}M\frac{\|H_{a}^{j}{\|}^{2}}{\Delta t}+(\frac{l_{I_{i}}}{2}\frac{\;\text{Trace}(\left(I_{3}-F_{a}^{j}\right)J_{d})}{\Delta t})-\Delta tV_{I_{i}}^{j} \end{array}$$ (7)

where $H_{a}^{j}={\left(O_{a}^{j}\right)}^{T}\Delta p_{a}^{j}$ and $J_{d}=\frac{\;\text{Trace}(J)}{2}I_{3}-J$.

The discrete action sum over the discretized time interval [0, *T*] = {*t*^0^, ⋯, *t*^*j*^|*t*^*j*^ = *t*^*j*−1^ + Δ*t*, *t*^0^ = 0, *t*^*V*^ = *T*}, is computed as follows.

$$\begin{array}{c} Y_{d}=\sum_{i=0}^{N+1}\sum_{j=1}^{V}L_{I_{i}}^{j} \end{array}$$

The discrete Lagrange–d’Alembert principle is

$$\begin{array}{c} \delta \sum_{i=0}^{N+1}\sum_{j=0}^{V}L^{j}+\sum_{j=0}^{V}\sum_{i=0}^{N+1}{{F_{d}}_{a_{i}}^{nc}}^{j}\cdot \delta \left(O_{a_{i}}^{j},p_{a_{i}}^{j}\right)=0 \end{array}$$ (8)

By applying the discrete Lagrange–d’Alembert principle (8), we get the discrete Euler–Lagrange equations for a node *a*_*i*_ in a compact form as

$$\begin{array}{c} \begin{array}{cc} & T_{e}^{*}L_{\left(F_{a_{i}}^{j-1},H_{a_{i}}^{j-1}\right)}(D_{F_{a_{i}}^{j-1}}L_{a_{i}}^{j-1},D_{H_{a_{i}}^{j-1}}L_{a_{i}}^{j-1})\\ & -Ad_{{\left(F_{a_{i}}^{j},H_{a_{i}}^{j}\right)}^{-1}}^{*}T_{e}^{*}L_{\left(F_{a_{i}}^{j},H_{a_{i}}^{j}\right)}(D_{F_{a_{i}}^{j}}L_{a_{i}}^{j},D_{H_{a_{i}}^{j}}L_{a_{i}}^{j})\\ & +T_{e}^{*}L_{\left(O_{a_{i}}^{j},p_{a_{i}}^{j}\right)}(D_{O_{a_{i}}^{j}}L_{a_{i}}^{j},D_{p_{a_{i}}^{j}}L_{a_{i}}^{j})+{\left(O_{a_{i}}^{j},p_{a_{i}}^{j}\right)}^{-1}{{F_{d}}_{a_{i}}^{nc}}^{j}=0 \end{array} \end{array}$$ (9)

Finally, using the definitions of adjoint and coadjoint actions, and cotangent lift of left translation which are presented in Appendix 6, Eqs (7) and (9) yields Eqs (10)–(12) and (14)–(16) to update rotations and positions of each node.

#### 3.1.1 Discrete Euler-Lagrange equations for rotations

- For the left node of the continuum manipulator (*a*_*i*=0_)
  $$\begin{array}{c} \begin{array}{c} {\left(F_{a_{0}}^{j}J_{d}-J_{d}{\left(F_{a_{0}}^{j}\right)}^{T}\right)}^{\vee }=-\frac{2\Delta t^{2}}{l_{I_{0}}}[\frac{1}{2}C_{1}\left(O_{a_{0}}^{T}\frac{\Delta p_{a_{0}}}{l_{I_{0}}}-E_{3}\right)\times O_{a_{0}}^{T}\Delta p_{a_{0}}\\ +\frac{1}{l_{I_{0}}}{\left({\left({\left(I+O_{a_{0+1}}^{T}O_{a_{0}}\right)}^{-1}\hat{C_{2}\psi_{a_{0}}}\left({\hat{\psi }}_{a_{0}}-2I\right)\right)}^{\left(A\right)}\right)}^{\vee }\\ -\Delta tO_{a_{0}}^{-1}N_{a_{0}}]{|}_{t=t^{j}}+{\left(J_{d}F_{a_{0}}^{j-1}-{\left(F_{a_{0}}^{j-1}\right)}^{T}J_{d}\right)}^{\vee } \end{array} \end{array}$$ (10)
- For the interior nodes of the continuum manipulator ∀*a*_*i*_, *i* ∈ {1, ⋯, *N* − 1}
  $$\begin{array}{c} \begin{array}{c} {\left(F_{a_{i}}^{j}J_{d}-J_{d}{\left(F_{a_{i}}^{j}\right)}^{T}\right)}^{\vee }=-\frac{{\Delta t}^{2}}{l_{I_{i}}}[\frac{1}{2}C_{1}(O_{a_{i}}^{T}\frac{\Delta p_{a_{i-1}}}{l_{I_{i}}}-E_{3})\times O_{a_{i}}^{T}\Delta p_{a_{i-1}}\\ +\frac{1}{2}C_{1}(O_{a_{i}}^{T}\frac{\Delta p_{a_{i}}}{l_{I_{i}}}-E_{3})\times O_{a_{i}}^{T}\Delta p_{a_{i}}+\frac{1}{l_{I_{i}}}{\left({\left({\left(I+O_{a_{i+1}}^{T}O_{a_{i}}\right)}^{-1}\hat{C_{2}\psi_{a_{i}}}({\hat{\psi }}_{a_{i}}-2I)\right)}^{\left(A\right)}\right)}^{\vee }\\ +\frac{1}{l_{I_{i}}}{\left({\left({\left(I+O_{a_{i-1}}^{T}O_{a_{i}}\right)}^{-1}\hat{C_{2}\psi_{a_{i-1}}}(-{\hat{\psi }}_{a_{i-1}}+2I)O_{a_{i-1}}^{T}O_{a_{i}}\right)}^{\left(A\right)}\right)}^{\vee }-\Delta tO_{a_{i}}^{-1}N_{a_{i}}]{|}_{t=t^{j}}\\ +{\left(J_{d}F_{a_{i}}^{j-1}-{\left(F_{a_{i}}^{j-1}\right)}^{T}J_{d}\right)}^{\vee } \end{array} \end{array}$$ (11)
- For the right node of the continuum manipulator (*a*_*i*=*N*_)
  $$\begin{array}{c} \begin{array}{c} {\left(F_{a_{N}}^{j}J_{d}-J_{d}{\left(F_{a_{N}}^{j}\right)}^{T}\right)}^{\vee }=-\frac{2{\Delta t}^{2}}{l_{I_{N}}}[\frac{1}{2}C_{1}(O_{a_{N}}^{T}\frac{\Delta p_{a_{N-1}}}{l_{I_{N}}}-E_{3})\times O_{a_{N}}^{T}\Delta p_{a_{N-1}}\\ +\frac{1}{l_{I_{N}}}{\left({\left({\left(I+O_{a_{N-1}}^{T}O_{a_{N}}\right)}^{-1}\hat{C_{2}\psi_{a_{N}-1}}({\hat{\psi }}_{a_{N-1}}-2I)\right)}^{\left(A\right)}\right)}^{\vee }\\ -\Delta tO_{a_{N}}^{-1}N_{a_{N}}]{|}_{t=t^{j}}+{\left(J_{d}F_{a_{N}}^{j-1}-{\left(F_{a_{N}}^{j-1}\right)}^{T}J_{d}\right)}^{\vee } \end{array} \end{array}$$ (12)

where the variable $\psi_{a_{i}}$ is defined as ${\hat{\psi }}_{a_{i}}≔{\text{exp}}^{-1}\left(O_{a_{i}}^{T}O_{a_{i+1}}\right)$ which is approximated by the Cayley transformation as ${\hat{\psi }}_{a_{i}}≔{\text{Cay}}^{-1}\left(O_{a_{i}}^{T}O_{a_{i+1}}\right)$, where The Cayley transformation and its inverse are defined in the following form for convenience $O_{a}^{T}O_{a+1}=\text{Cay}\left({\hat{\psi }}_{a}\right)=\frac{I+{\hat{\psi }}_{a}}{I-{\hat{\psi }}_{a}}$ with inverse ${\hat{\psi }}_{a}={\text{Cay}}^{-1}\left(O_{a}^{T}O_{a+1}\right)=2\frac{O_{a}^{T}O_{a+1}-I}{O_{a}^{T}O_{a+1}+I}$ (see, [35, 42]). In addition, $\Delta p_{a_{i}}{|}_{t=t^{j}}=p_{a_{i}}^{j+1}-p_{a_{i}}^{j}$.

For discrete Euler-Lagrange equations for rotations, Eqs (10)–(12), one has to solve an implicit expression of the form

$$\begin{array}{c} \hat{U}=F_{a}J_{d}-J_{d}F_{a}^{T},\;\forall a\in \left\{a_{0},\cdots ,a_{N}\right\} \end{array}$$ (13)

In order to solve Eq (13) for *F* ∈ *SO*(3), (the vector U or the right hand sides of Eqs (10)–(12) and the symmetric non-standard inertia matrix *J*_*d*_ are known), a Newton iteration method based on the Cayley transformation is applied (as described in [39], Section 3:3:8]).

#### 3.1.2 Discrete Euler-Lagrange equations for translations

- For the left node of the continuum manipulator (*a*_*i*=0_)
  $$\begin{array}{c} \begin{array}{c} p_{a_{0}}^{j+1}=\frac{2{\Delta t}^{2}}{l_{I_{0}}M}[\frac{1}{2}O_{a_{0}}C_{1}(O_{a_{0}}^{T}\frac{\Delta p_{a_{0}}}{l_{I_{0}}}-E_{3})+\frac{1}{2}O_{a_{0+1}}C_{1}(O_{a_{0+1}}^{T}\frac{\Delta p_{a_{0}}}{l_{I_{0}}}-E_{3})\\ -\frac{l_{I_{0}}}{2}F_{a_{0}}^{c}-\Delta tO_{a_{0}}^{-1}F_{a_{0}}]{|}_{t=t^{j}}+2p_{a_{0}}^{j}+p_{a_{0}}^{j-1} \end{array} \end{array}$$ (14)
- For the interior nodes of the continuum manipulator ∀*a*_*i*_, *i* ∈ {1, ⋯, *N* − 1}
  $$\begin{array}{c} \begin{array}{c} p_{a_{i}}^{j+1}=\frac{{\Delta t}^{2}}{l_{I_{i}}M}[\frac{1}{2}O_{a_{i}}C_{1}(O_{a_{i}}^{T}\frac{\Delta p_{a_{i}}}{l_{I_{i}}}-E_{3})-\frac{1}{2}O_{a_{i-1}}C_{1}(O_{a_{i-1}}^{T}\frac{\Delta p_{a_{i-1}}}{l_{I_{i}}}-E_{3})\\ +\frac{1}{2}O_{a_{i+1}}C_{1}(O_{a_{i+1}}^{T}\frac{\Delta p_{a_{i}}}{l_{I_{i}}}-E_{3})-\frac{1}{2}O_{a_{i}}C_{1}(O_{a_{i}}^{T}\frac{\Delta p_{a_{i-1}}}{l_{I_{i}}}-E_{3})\\ -\frac{l_{I_{i}}}{2}F_{a_{i}}^{c}-\Delta tO_{a_{i}}^{-1}F_{a_{i}}]{|}_{t=t^{j}}+2p_{a_{i}}^{j}+p_{a_{i}}^{j-1} \end{array} \end{array}$$ (15)
- For the right node of the continuum manipulator (*a*_*i*=*N*_)
  $$\begin{array}{c} \begin{array}{c} p_{a_{N}}^{j+1}=\frac{2{\Delta t}^{2}}{l_{I_{N}}M}[-\frac{1}{2}O_{a_{N-1}}C_{1}(O_{a_{N-1}}^{T}\frac{\Delta p_{a_{N-1}}}{l_{I_{N}}}-E_{3})-\frac{1}{2}O_{a_{N}}C_{1}(O_{a_{N}}^{T}\frac{\Delta p_{a_{N-1}}}{l_{I_{N}}}-E_{3})\\ -\frac{l_{I_{N}}}{2}F_{a_{N}}^{c}-\Delta tO_{a_{N}}^{-1}F_{a_{N}}]{|}_{t=t^{j}}+2p_{a_{N}}^{j}+p_{a_{N}}^{j-1} \end{array} \end{array}$$ (16)

**Remark 1** *For magnetic actuation, we fabricate manipulators with embedded permanent magnets. Consider Magnet i with weight m*_*i*_ *in an interval* $I_{i}$ *in which a*_*i*_ *and a*_*i*+1_, *i* ∈ {0, ⋯, *N* − 1} *are considered as left and right nodes of the interval. Therefore, the distributed load per unit length for Nodes a*_*i*_ *and a*_*i*+1_ *are approximately considered as* $M+\frac{m_{i}}{2l_{I_{i}}}$. *In addition, if Magnet i is embedded at the tip*, $M+\frac{m_{i}}{l_{I_{N}}}$ *replaces the distributed load per unit length of Node a*_*N*+1_ *while the distributed load per unit length of Node a*_*N*_ *is unchanged*.

### 3.2 Estimation

**Fig 3:**
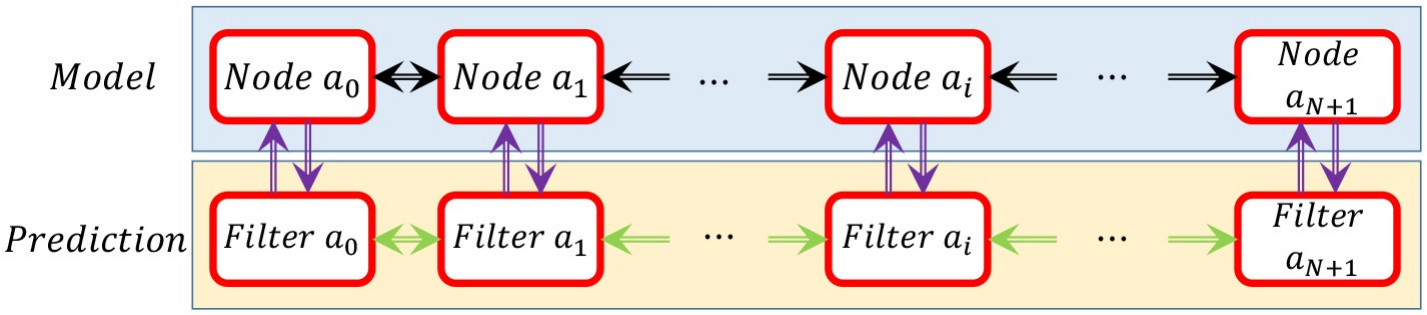
Configuration of the nodes of the model and the corresponding distributed filters. Filter *a*_*i*_ and Node *a*_*i*_ are coupled with the adjacent nodes in succession.

In this section, online distributed estimation algorithms are developed to predict the model dissipation error. The structure of the estimator mimics the model’s structure, as explained in Section 3.1. To design the estimation protocol, we follow the same line of ideas as in [42] but in distributed multi-systems configuration. We consider each node as an individual system coupled with the other adjacent nodes, i.e., neighbors, in succession. In other words, each node exchanges its local pose (position and orientation) with its neighbors. It should be noted that the estimation filters are designed and implemented for each node. Fig 3 shows the configuration of the distributed filters and nodes.

For simplicity, we assume that each node’s position is included in its state vector. Therefore, given node *a*_*i*_, *i* = {0, ⋯, *N*}, the time-varying dynamic equations based on Eqs (14)–(16) can be written as

$$\begin{array}{c} \begin{array}{cc} S_{a_{i}}^{j+1} & =F_{a_{i}}\left(S_{a_{i}}^{j},U_{a_{i}}^{j},j\right)+G_{a_{i}}^{j}\left(S_{a_{i}}^{j}\right)F_{a_{i}}^{j},\\ Y_{a_{i}}^{j+1} & =H_{a_{i}}^{j+1}S_{a_{i}}^{j+1}, \end{array} \end{array}$$ (17)

where *j* = {1, 2, ⋯}, *i* = {0, 2, ⋯, *N*}, $S_{a_{i}}^{j}={\left[p_{a_{i}}^{j-1}\;p_{a_{i}}^{j}\right]}^{T}\in R^{6}$ is the true state vector, $U_{a_{i}}^{j}$ is a known input vector, $F_{a_{i}}\in R^{6}$ is sufficiently differentiable, $G_{a_{i}}^{j}={\left[0_{3\times 3},\frac{-2{\Delta t}^{3}}{l_{I_{i}}M}{O_{a_{i}}^{j}}^{-1}\right]}^{T}\in R^{6\times 3}$ is the model dissipation error matrix and in the considered systems is time-varying, $F_{a_{i}}^{j}\in R^{3}$ is a modified viscous model dissipation force or hysteretic damping force in the form of $K_{a_{i}}^{j}\circ \Delta t^{-1}\left(p_{a_{i}}^{j}-p_{a_{i}}^{j-1}\right)$, where ∘ denotes Hadamard product and $K_{a_{i}}^{j}\in R^{3}$ is damping capacity that is independent of frequency of motion and needs to be estimated, $H_{a_{i}}^{j+1}\in R^{3\times 6}$ is the output matrix, and $Y_{a_{i}}^{j+1}\in R^{3\times 1}$ is the output vector.

By substituting $F_{a_{i}}^{j}=K_{a_{i}}^{j}\circ \Delta t^{-1}\left(p_{a_{i}}^{j}-p_{a_{i}}^{j-1}\right)$ into Eq (17), one has

$$\begin{array}{c} \begin{array}{cc} S_{a_{i}}^{j+1} & =F_{a_{i}}\left(S_{a_{i}}^{j},U_{a_{i}}^{j},j\right)+G_{a_{i}}^{j}\left(S_{a_{i}}^{j}\right)K_{a_{i}}^{j}\circ \Delta t^{-1}\left(p_{a_{i}}^{j}-p_{a_{i}}^{j-1}\right),\\ Y_{a_{i}}^{j+1} & =H_{a_{i}}^{j+1}S_{a_{i}}^{j+1}, \end{array} \end{array}$$ (18)

Using commutative property of Hadamard product, we can write $G_{a_{i}}^{j}\left(S_{a_{i}}^{j}\right)K_{a_{i}}^{j}\circ \Delta t^{-1}\left(p_{a_{i}}^{j}-p_{a_{i}}^{j-1}\right)=G_{a_{i}}^{j}\left(S_{a_{i}}^{j}\right)\Delta t^{-1}\left(p_{a_{i}}^{j}-p_{a_{i}}^{j-1}\right)\circ K_{a_{i}}^{j}$. Then, Hadamard product can be converted to matrix multiplication by the corresponding diagonal matrix of the vector $G_{a_{i}}^{j}\left(S_{a_{i}}^{j}\right)\Delta t^{-1}\left(p_{a_{i}}^{j}-p_{a_{i}}^{j-1}\right)$ which is denoted by $G_{a_{i}}^{j}=G_{a_{i}}^{j}\left(S_{a_{i}}^{j}\right)\Delta t^{-1}\text{diag}(p_{a_{i}}^{j}-p_{a_{i}}^{j-1})$ and $G_{a_{i}}^{j}\in R^{6\times 3}$. Therefore, Eq (18) may be written as

$$\begin{array}{c} \begin{array}{cc} S_{a_{i}}^{j+1} & =F_{a_{i}}\left(S_{a_{i}}^{j},U_{a_{i}}^{j},j\right)+G_{a_{i}}^{j}\left(S_{a_{i}}^{j}\right)K_{a_{i}}^{j},\\ Y_{a_{i}}^{j+1} & =H_{a_{i}}^{j+1}S_{a_{i}}^{j+1}, \end{array} \end{array}$$

**Fig 4:**
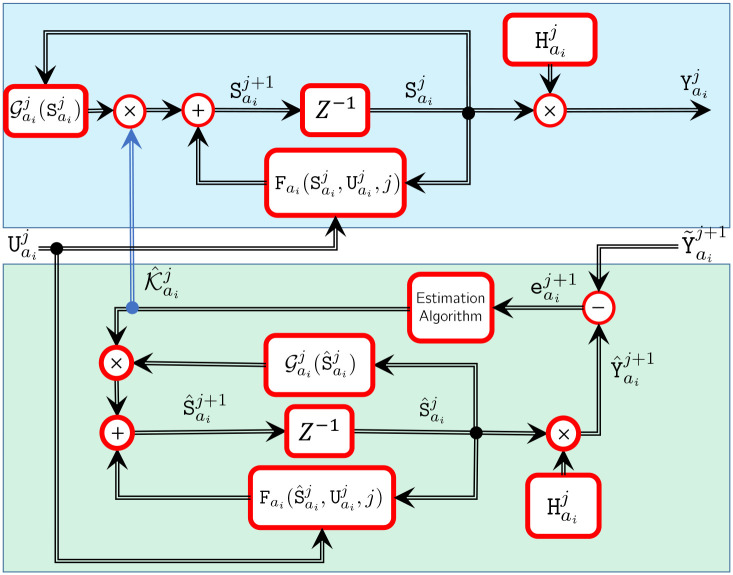
Block diagram of the proposed prediction filter *a*_*i*_ coupled with the node *a*_*i*_’s model. The filter employs the model’s output to perform a pointwise optimization problem to predict the damping capacity ${\hat{K}}_{a_{i}}^{i}$.

Estimation of the state and output vector is given by

$$\begin{array}{c} \begin{array}{cc} {\hat{S}}_{a_{i}}^{j+1} & =F_{a_{i}}\left({\hat{S}}_{a_{i}}^{j},U_{a_{i}}^{j},j\right)+G_{a_{i}}^{j}\left({\hat{S}}_{a_{i}}^{j}\right){\hat{K}}_{a_{i}}^{j},\\ {\hat{Y}}_{a_{i}}^{j+1} & =H_{a_{i}}^{j+1}{\hat{S}}_{a_{i}}^{j+1}, \end{array} \end{array}$$

where ${\hat{S}}_{a_{i}}^{j}={\left[{\hat{p}}_{a_{i}}^{j-1}\;{\hat{p}}_{a_{i}}^{j}\right]}^{T}\in R^{6}$ is the estimation of the state vector, ${\hat{K}}_{a_{i}}^{j}\in R^{3}$ is the model dissipation error estimates, ${\hat{Y}}_{a_{i}}^{j+1}\in R^{3\times 1}$ is the output vector estimates. Finally, ${\tilde{Y}}_{a_{i}}^{j+1}$ denotes the measurement. The block diagram of the filter integrated with the model is shown in Fig 4. To find ${\hat{K}}_{a_{i}}^{j}$ for the node *a*_*i*_ at the time *j*, we consider a pointwise cost function that penalizes and minimizes the estimation error vector (the error between measurement and the output estimation) at the next sampling time *j* + 1, and estimated damping capacity $K_{a_{i}}^{j+1}$. The cost function for each node *a*_*i*_ is given as

$$\begin{array}{c} J_{a_{i}}\left(K_{a_{i}}^{j+1}\right)=\frac{1}{2}{e_{a_{i}}^{j+2}}^{T}Re_{a_{i}}^{j+2}+\frac{1}{2}{K_{a_{i}}^{j+1}}^{T}WK_{a_{i}}^{j+1} \end{array}$$ (19)

where $e_{a_{i}}^{j+2}={\hat{Y}}_{a_{i}}^{j+2}-{\tilde{Y}}_{a_{i}}^{j+2}$, $W\in R^{3\times 3}$, and $R\in R^{3\times 3}$ are positive semi-definite and positive definite matrices, respectively.

In order to derive an optimal estimation law, we need to approximate the output estimation vector ${\hat{Y}}_{a_{i}}^{j+2}$ at the next sampling time *j* + 2, which is given by its Taylor series expansion as follows

$$\begin{array}{c} {\hat{Y}}_{a_{i}}^{j+2}\approx {\hat{Y}}_{a_{i}}^{j+1}+Z\left({\hat{S}}_{a_{i}}^{j+1},\Delta t\right)+\Lambda \left(\Delta t\right)M\left({\hat{S}}_{a_{i}}^{j+1}\right){\hat{K}}_{a_{i}}^{j+1} \end{array}$$ (20)

where

$$\begin{array}{c} \begin{array}{cc} Z({\hat{S}}_{a_{i}}^{j+1},j) & =\Delta tL_{F_{a_{i}}}^{1}\left(H_{a_{i}}^{j+1}{\hat{S}}_{a_{i}}^{j+1}\right)=\Delta t\frac{\partial H_{a_{i}}^{j+1}{\hat{S}}_{a_{i}}^{j+1}}{\partial {\hat{S}}_{a_{i}}^{j+1}}F_{a_{i}}\\ \Lambda (\Delta t) & =\Delta tI_{3}\\ M({\hat{S}}_{a_{i}}^{j+1}) & =\frac{-2{\Delta t}^{2}}{l_{I_{i}}M}{O_{a_{i}}^{j}}^{-1}\text{diag}(p_{a_{i}}^{j}-p_{a_{i}}^{j-1}) \end{array} \end{array}$$

Similarly, we may expand the *i*^*th*^ component of ${\tilde{Y}}_{a_{i}}^{j+2}$ in an first-order Taylor series so that

$$\begin{array}{c} {\tilde{Y}}_{a_{i}}^{j+2}\approx {\tilde{Y}}_{a_{i}}^{j+1}+d_{a_{i}}^{j+1} \end{array}$$

where the *h*^*th*^ component of $d_{a_{i}}^{j+1}\in R^{3}$ is

$$\begin{array}{c} {d_{a_{i}}^{j+1}}_{h}={{\tilde{Y}}_{a_{i}}^{j+1}}_{h}-{{\tilde{Y}}_{a_{i}}^{j}}_{h} \end{array}$$

Solving Eq (19) for ${\hat{K}}_{a_{i}}^{j+1}$ by considering Eq (20) yields

$$\begin{array}{c} \begin{array}{cc} {\hat{K}}_{a_{i}}^{j+1}=\{{\left[\Lambda \left(\Delta t\right)M\left({\hat{S}}_{a_{i}}^{j+1}\right)\right]}^{T}R\left[\Lambda \left(\Delta t\right)M\left({\hat{S}}_{a_{i}}^{j+1}\right)\right]+W{\}}^{-1} & \times {\left[\Lambda \left(\Delta t\right)M\left({\hat{S}}_{a_{i}}^{j+1}\right)\right]}^{T}R\\ & \times [Z\left({\hat{S}}_{a_{i}}^{j+1},j\right)+e_{a_{i}}^{j+1}-d_{a_{i}}^{j+1}] \end{array} \end{array}$$ (21)

Stability and convergence analysis of the filters can be found in [42]. Here we skip the analysis for brevity.

## 4 Simulation and experimental results

In this section, we investigate and analyze the solver’s performance with different continuum manipulators through experiments. The experiments here are expected to provide validation of the theoretical formulation for a variety of scenarios. As discussed earlier, it is worth remembering that the dynamic equations for translation and rotation are decoupled. Eqs (14)–(16) can be solved explicitly to update nodes translation, while an iterative method—as it is discussed in Section 3.1.1—is necessary to solve Eqs (10)–(12) for updating the rotations. It should be pointed out that the estimation law (21) is implemented for every node to estimate conservative forces. The required parameters for the simulation will be discussed for each experiment.

### 4.1 Flexible metal rods

**Fig 5:**
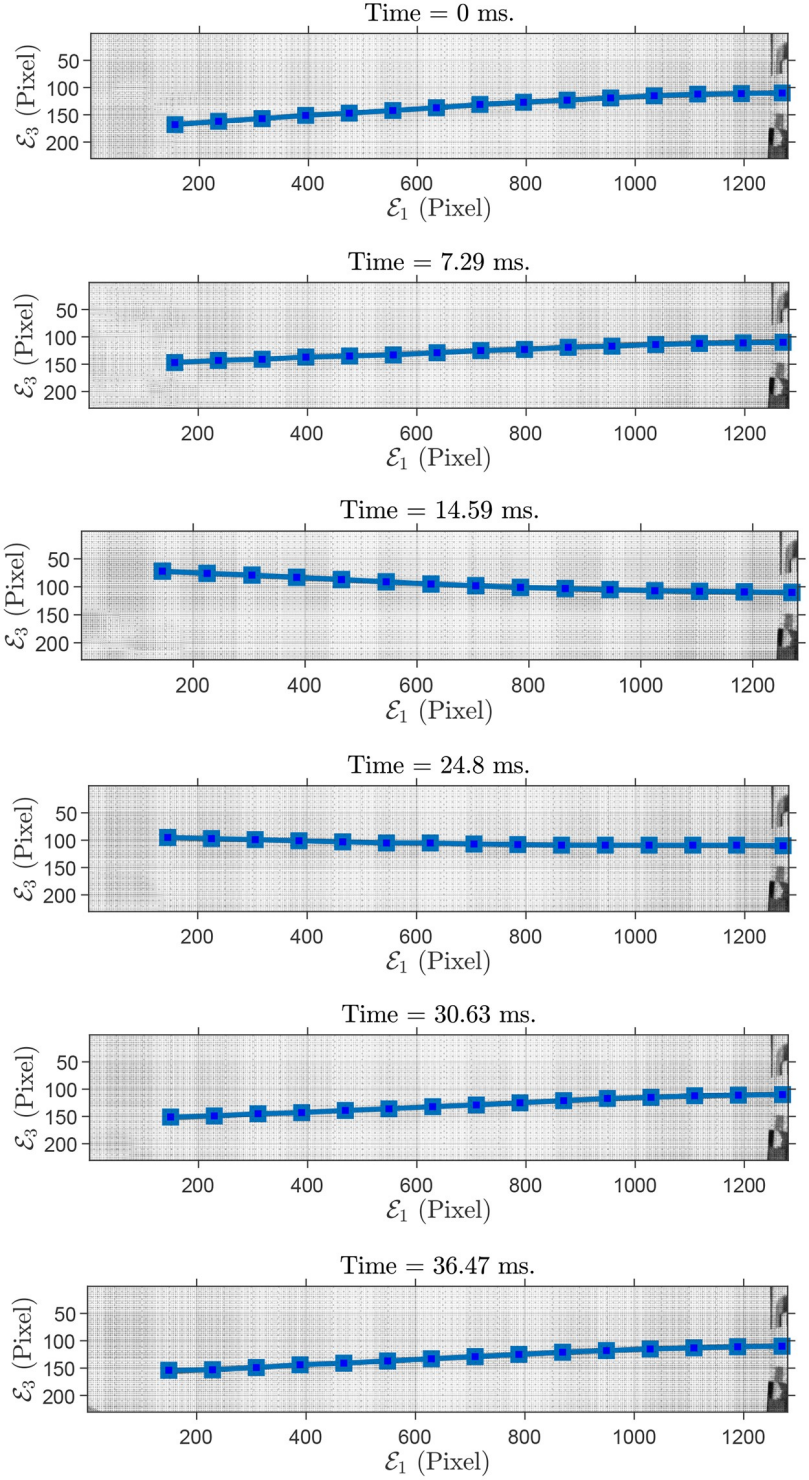
Sample of grabbed images of flexible rod (AlSi05) configurations, in-plane experiment ($E_{1}E_{3}$-plane). 15 discretization nodes, depicted in blue squares, are superimposed on the flexible rod.

**Fig 6:**
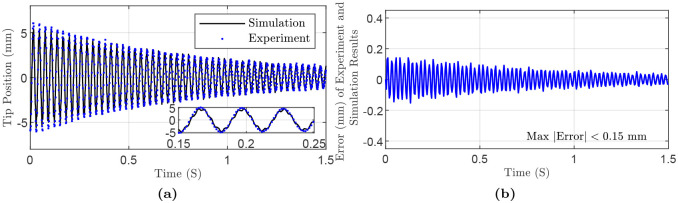
Simulation and experiment results for flexible rod (AlSi05), in-plane experiment: (a) Tip position in $E_{1}$-axis direction. Inset highlights the results in a small time range. (b) Error in $E_{1}$-axis direction.

As a first case, we consider a cylindrical rod made of aluminum (Al4043/ AlSi5) with diameter of 2 mm, length 200 mm, mass density 2690 $\frac{\text{kg}}{m^{3}}$, Young’s modulus 75 GPa and Poisson’s ratio 0.33. As a first example, we suppose a planar motion of the rod in the $E_{1}E_{3}$-plane with the initial deflection $\theta_{E_{1}E_{3}}=3.69^{}$. $\theta_{E_{1}E_{3}}$ denotes the rotation of the tip around $E_{1}$ in the $E_{1}E_{3}$-plane. It is worth pointing out that the nondissipative force is the gravity in the $E_{3}$-axis direction. In addition, we run a simulation with the given specifications with *N* = 15 discretization nodes. These points are depicted in Fig 5 together with some time-evolved configurations of the rod. For simplicity, only tip positions are used for the comparison with the simulation results. The maximum and mean absolute error are 0.15 mm (i.e., 2.5% of displacement), and 0.05 mm, respectively. The error, simulation and experiment results are shown in Fig 6 and the simulation parameters are summarized in Table 3.

**Table 3:** Simulation parameters in Eqs (10)–(16) and (21) for in-plane experiment of flexible cylindrical rod (AlSi05).

M	$8.45\times 10^{-3}\;\frac{g}{\text{mm}}$
Number of elements	15
$l_{I_{i}}{|}_{i=\{1,2,\cdots ,N\}}$	$\frac{200}{15}\;\text{mm}$
*J*_*d*_	diag(0, 2.11, 2.11) g × mm^2^
$E_{3}$	[1, 0, 0]^*T*^
$F_{a_{i}}^{c}{|}_{i=\{1,2,\cdots ,N+1\}}$	${\left[0,0,8.29\right]}^{T}\times 10^{4}\;\frac{\text{g}}{{\text{S}}^{2}}$
*C*_1_	diag(2.35, 0.88, 0.88) × 10^14^
*C*_2_	diag(4.42, 5.89, 5.89) × 10^13^
Time step	1 × 10^−6^
Simulation time	1.5 (S)
R	*I*_3_ × 10^4^
W	*I*_3_ × 10^−1^

**Fig 7:**
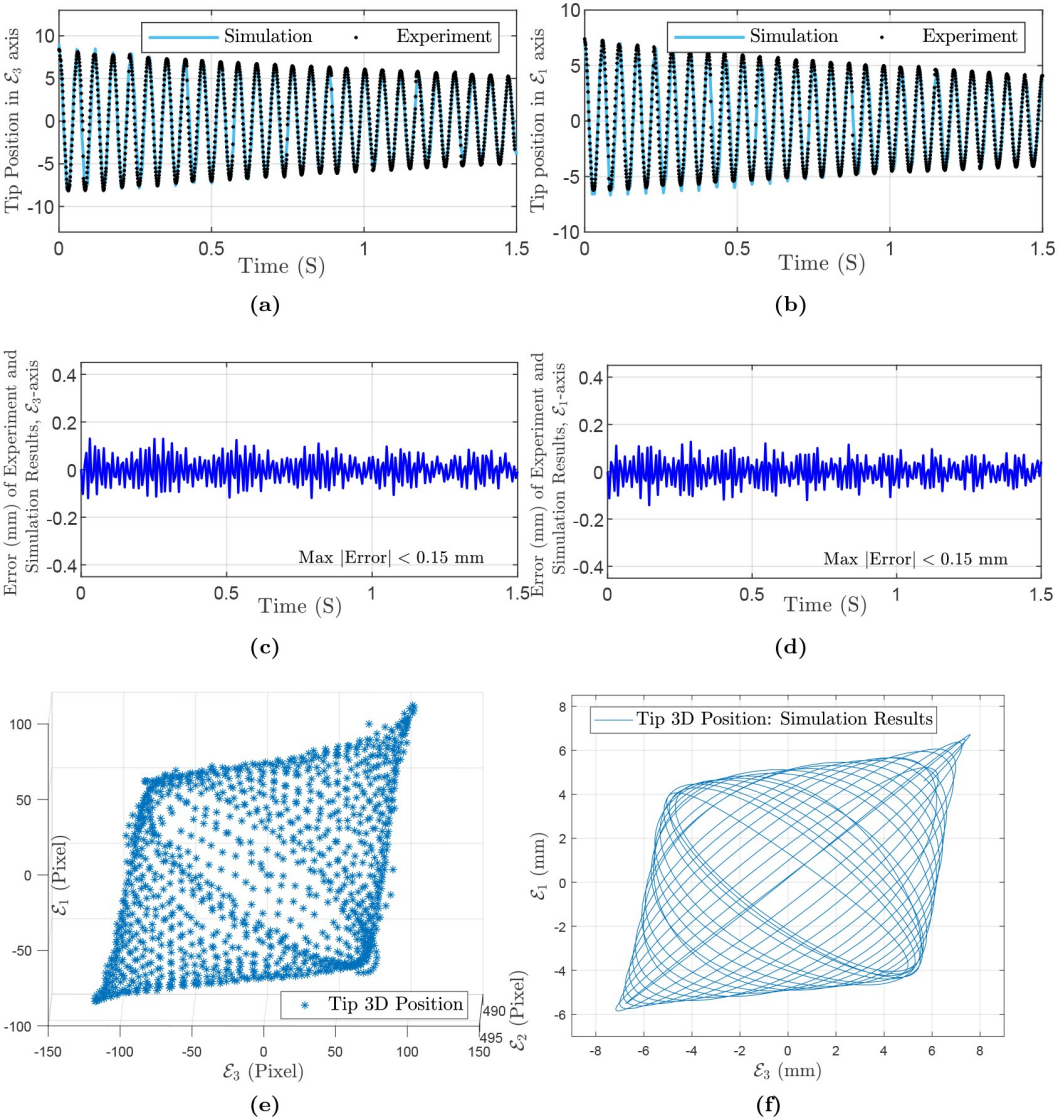
Simulation and experiment results for flexible rod (AlSi05), out-of-plane experiment: (a) Tip position in $E_{3}$-axis direction. (b) Tip position in $E_{1}$-axis direction. (c) Error in $E_{3}$-axis direction. (d) Error in $E_{1}$-axis direction. (e) Tip 3D position: non-planar experiment. (f) Tip 3D position: simulation.

Next, we consider a three-dimensional motion for a rod with the same material as the first case but with diameter *d* = 1 mm with initial deflections $\theta_{E_{1}E_{2}}=-5.53^{}$ (i.e., the tip distance is 8 mm from $E_{2}$-axis) and $\theta_{E_{1}E_{3}}=6.52^{}$ (i.e., the tip distance is 10 mm from $E_{3}$-axis) in the $E_{3}E_{2}$ and $E_{1}E_{2}$ planes, respectively. The results of the experiment, simulation, and error are depicted in Fig 7. Maximum and mean absolute error in both $E_{3}$ and $E_{1}$ axes are 0.15 mm (i.e., 2.12%), and 0.05 mm, respectively. The simulation parameters are summarized in Table 4.

**Table 4:** Simulation parameters in Eqs (10)–(16) and (21) for out-of-plane experiment of flexible cylindrical rod (AlSi05).

M	$2.11\times 10^{-3}\;\frac{g}{\text{mm}}$
Number of elements	15
$l_{I_{i}}{|}_{i=\{1,2,\cdots ,N\}}$	$\frac{200}{15}\;\text{mm}$
*J*_*d*_	diag(0, 0.13, 0.13) g × mm^2^
$E_{3}$	[1, 0, 0]^*T*^
$F_{a_{i}}^{c}{|}_{i=\{1,2,\cdots ,N+1\}}$	${\left[0,0,2.07\right]}^{T}\times 10^{4}\;\frac{\text{g}}{{\text{S}}^{2}}$
*C*_1_	diag(5.89, 2.21, 2.21) × 10^13^
*C*_2_	diag(2.76, 3.68, 3.68) × 10^12^
Time step	1 × 10^−6^
Simulation time	1.5 (S)
R	*I*_3_ × 10^4^
W	*I*_3_ × 10^−1^

### 4.2 Polymer-based rods

**Fig 8:**
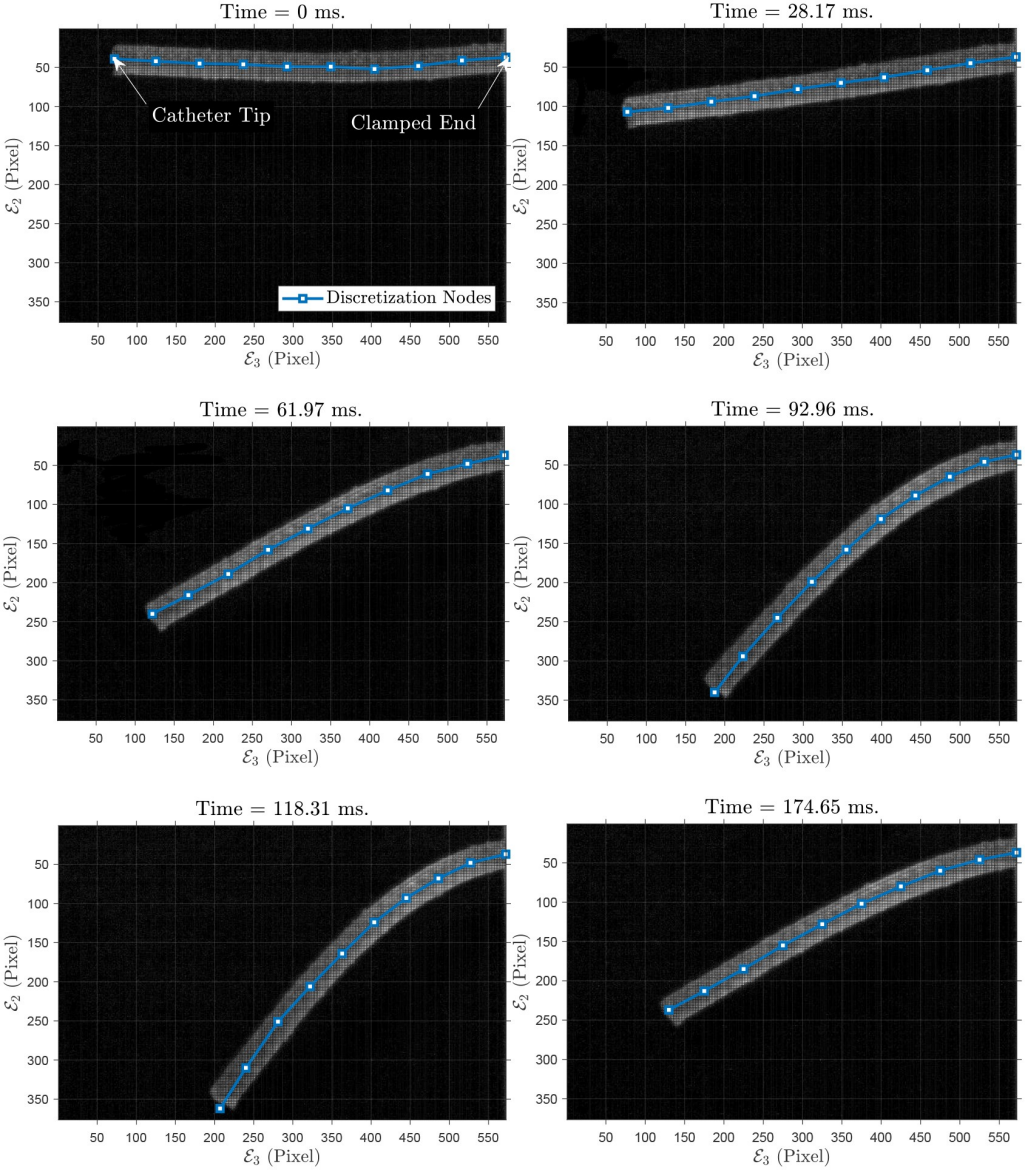
Sample of grabbed images for Polydimethylsiloxane (PDMS) rod without any embedded magnet in 2D experiment: In-plane motion. Also, 10 discretization points are superimposed on the soft rod.

**Fig 9:**
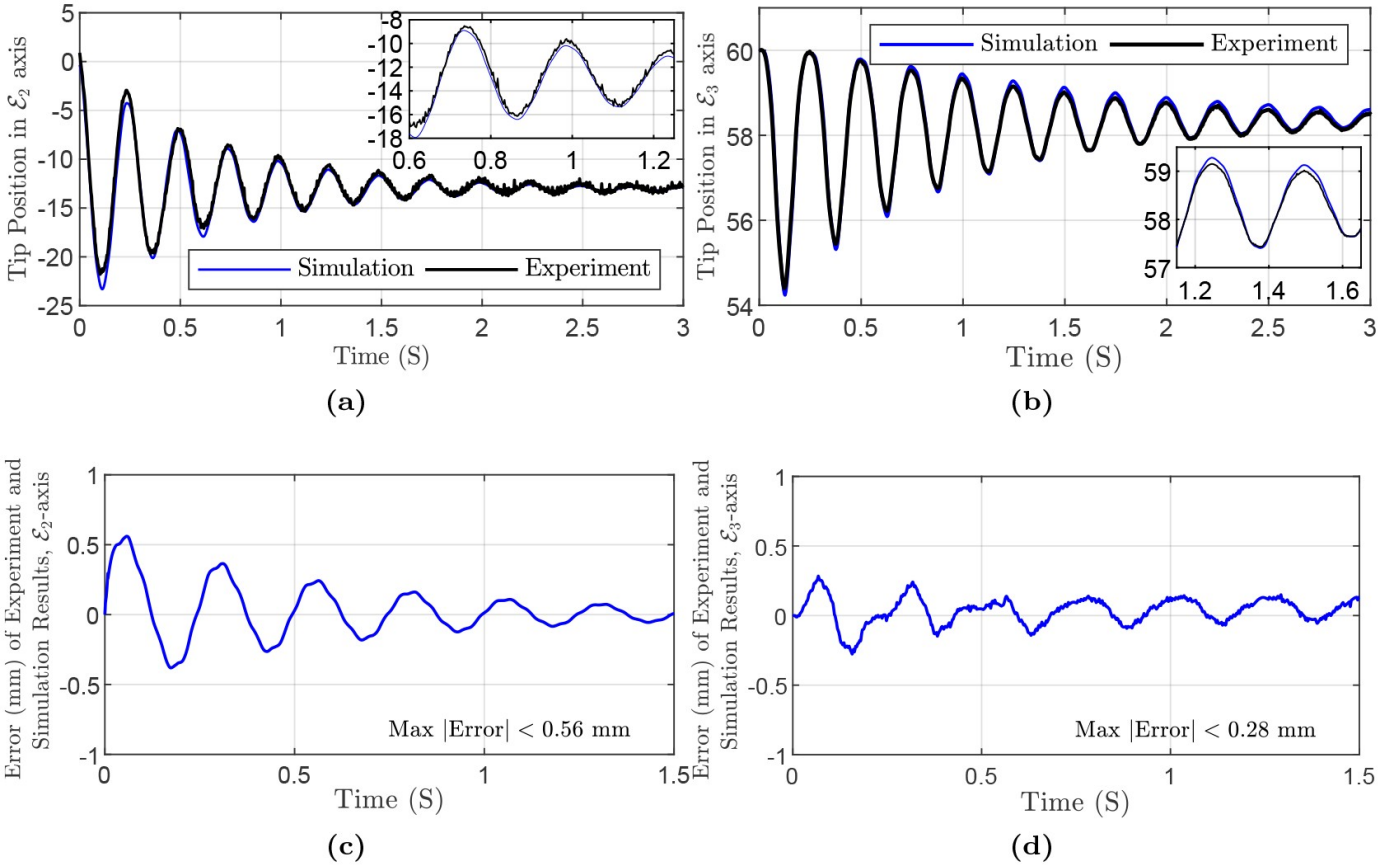
Simulation and experiment results of Polydimethylsiloxane (PDMS) rod, planar motion: (a) Tip Position in $E_{2}$-axis direction. Inset magnifies the results in a small time range. (b) Tip Position in $E_{3}$-axis direction. (c) Error in $E_{2}$-axis direction. (d) Error in $E_{3}$-axis direction.

In the second experiment, a cylindrical Polydimethylsiloxane (PDMS) rod is considered. Fig 8 depicts the rod, which has diameter *D* = 5 mm, length *L* = 60.5 mm. In addition, for the rod, mass density $\rho =1101\;\frac{\text{kg}}{m^{3}}$, Young’s modulus *E* = 365.12 MPa, and Poisson’s ratio *ν* = 0.5. In this experiment, the rod is kept straight initially, with the gravity acting along $E_{2}$, Fig 8(a). Experiment and simulation results for the tip position and the error are depicted in Fig 9. Also, maximum and mean absolute error in $E_{2}$-axis are 0.56 mm (i.e., 4.87%), and 0.05 mm, respectively. In $E_{3}$-axis, maximum and mean absolute error are 0.28 mm (i.e., 4.89%), and 0.05 mm, respectively. The simulation parameters are summarized in Table 5.

**Table 5:** Simulation parameters in Eqs (10)–(16) and (21) for in-plane experiment of PDMS rod (without magnet).

M	$21.62\times 10^{-3}\;\frac{g}{\text{mm}}$
Number of elements	10
$l_{I_{i}}{|}_{i=\{1,2,\cdots ,N\}}$	$\frac{60.5}{10}\;\text{mm}$
*J*_*d*_	diag(0, 33.78, 33.78) g × mm^2^
$E_{3}$	[1, 0, 0]^*T*^
$F_{a_{i}}^{c}{|}_{i=\{1,2,\cdots ,N+1\}}$	${\left[0,0,2.12\right]}^{T}\times 10^{5}\;\frac{\text{g}}{{\text{S}}^{2}}$
*C*_1_	diag(7.17, 2.39, 2.39) × 10^9^
*C*_2_	diag(0.75, 1.12, 1.12) × 10^10^
Time step	8 × 10^−5^
Simulation time	1.5 (S)
R	*I*_3_ × 10^5^
W	*I*_3_ × 10^−1^

**Fig 10:**
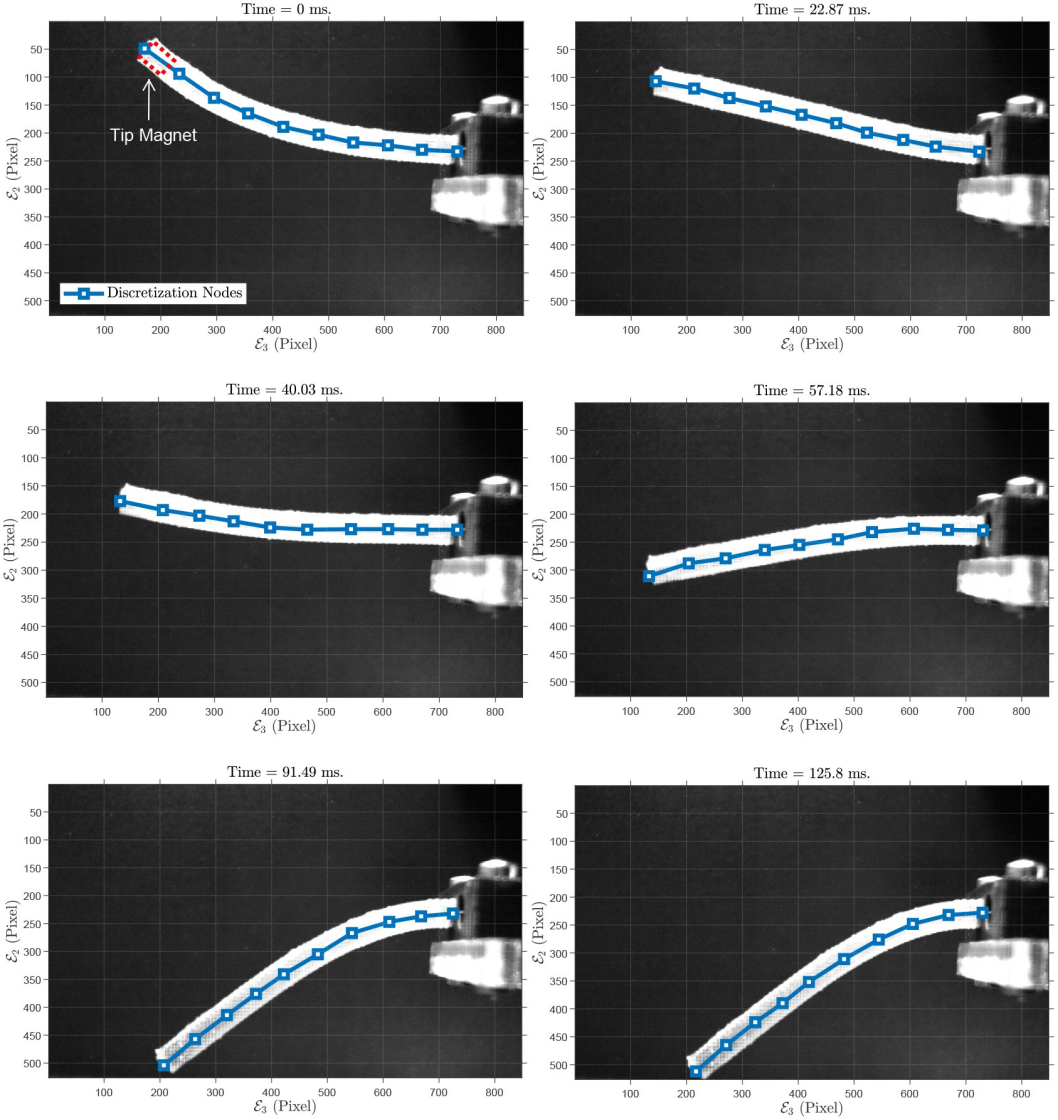
Sample of grabbed images: Polydimethylsiloxane (PDMS) rod with an embedded magnet at the tip in 2D experiment: Motion in a plane. Also, 10 discretization points are shown on the soft rod.

**Fig 11:**
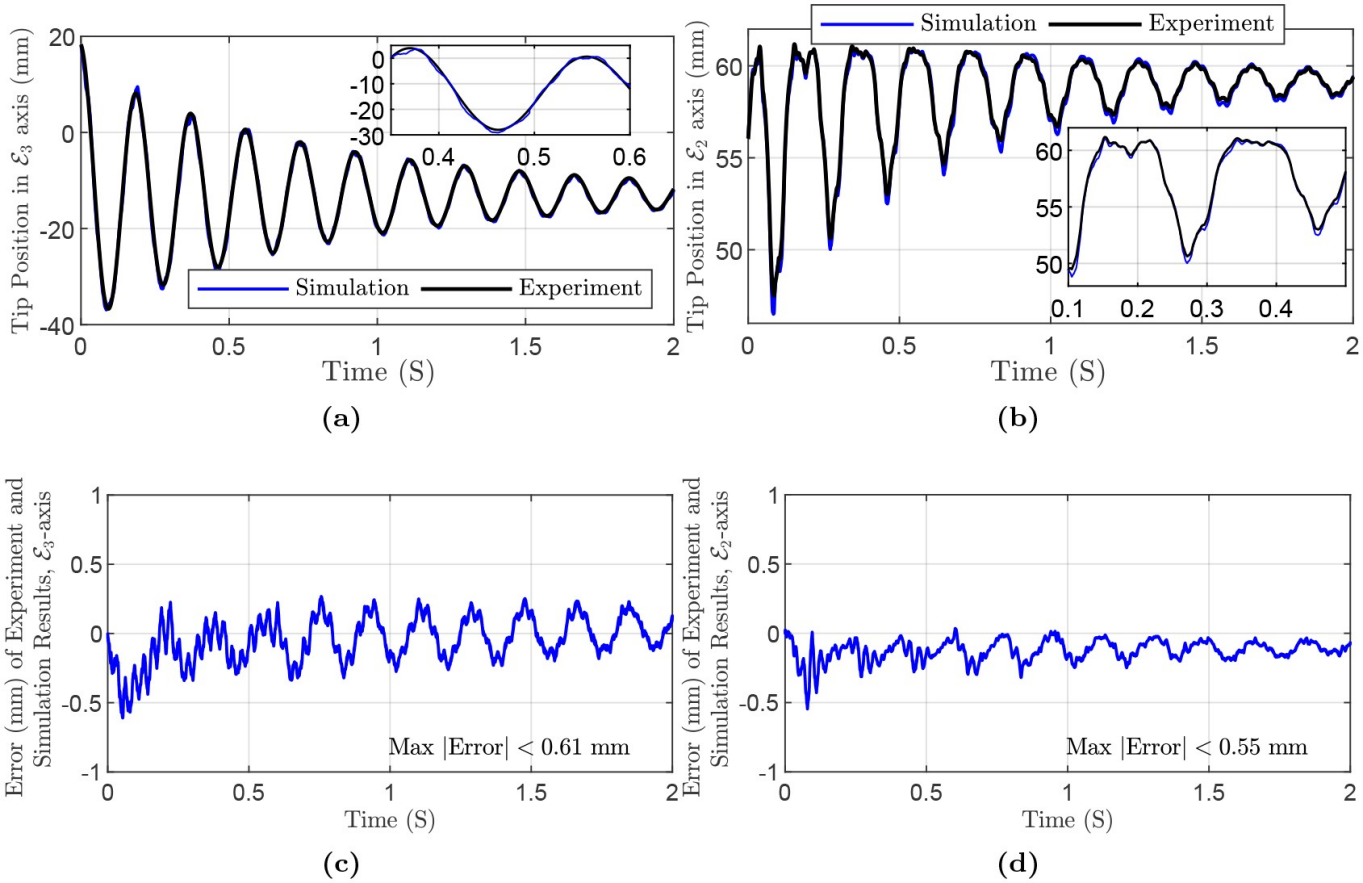
Simulation and experiment results of Polydimethylsiloxane (PDMS) rod with an embedded magnet at the tip, planar experiment: (a) Tip Position in $E_{3}$-axis direction. Inset highlights the results in a small time range. (b) Tip Position in $E_{2}$-axis direction. (c) Error in $E_{3}$-axis direction. (d) Error in $E_{2}$-axis direction.

For the next experiment, we fabricated a cylindrical PDMS manipulator with a permanent magnet at the tip. The initial and some time-evolved configurations of the rod are depicted in Fig 10. The specifications of the rod are as follows: diameter *D* = 4 mm, length *L* = 60.55 mm. In addition, the embedded neodymium magnet is a cylindrical magnet with diameter *D*_*m*_ = 2 mm, height *L*_*m*_ = 4 mm, mass *M*_*m*_ = 9.6 × 10^−5^ kg. The rod moves around $E_{1}$ with the initial deflection $\theta_{E_{2}E_{3}}=-41.74$ in the $E_{2}E_{3}$-plane. Also, maximum and mean absolute error in $E_{3}$-axis are 0.55 mm (i.e., 1.16%), and 0.13 mm, respectively. In $E_{2}$-axis, maximum and mean absolute error are 0.61 mm (i.e., 3.35%), and 0.14 mm, respectively. Tip positions in the experiment, simulation and the error are shown in Fig 11. The simulation parameters are summarized in Table 6.

**Table 6:** Simulation parameters in Eqs (10)–(16) and (21) for in-plane and circular-motion experiments of PDMS rod (with tip magnet).

M	$13.83\times 10^{-3}\;\frac{g}{\text{mm}}$
Number of elements	10
$l_{I_{i}}{|}_{i=\{1,2,\cdots ,N\}}$	$\frac{60.55}{10}\;\text{mm}$
*J*_*d*_	diag(0, 13.83, 13.83) g × mm^2^
$E_{3}$	[1, 0, 0]^*T*^
$F_{a_{i}}^{c}{|}_{i=\{1,2,\cdots ,N\}}$	${\left[0,0,1.357\right]}^{T}\times 10^{5}\;\frac{\text{g}}{{\text{S}}^{2}}$
$F_{a_{N+1}}^{c}$	${\left[0,0,1.36\right]}^{T}\times 10^{5}\;\frac{\text{g}}{{\text{S}}^{2}}$
*C*_1_	diag(4.58, 1.53, 1.53) × 10^9^
*C*_2_	diag(3.06, 4.59, 4.59) × 10^9^
Time step	9 × 10^−5^
Simulation time	2 (S)
R	*I*_3_ × 10^5^
W	*I*_3_ × 10^−1^
Magnet weight	0.096 g

Hereafter, a magnetic field generation setup is employed to actuate the manipulators. The following section introduces magnetic field generation setup and the related background.

### 4.3 Magnetic field generation

**Fig 12:**
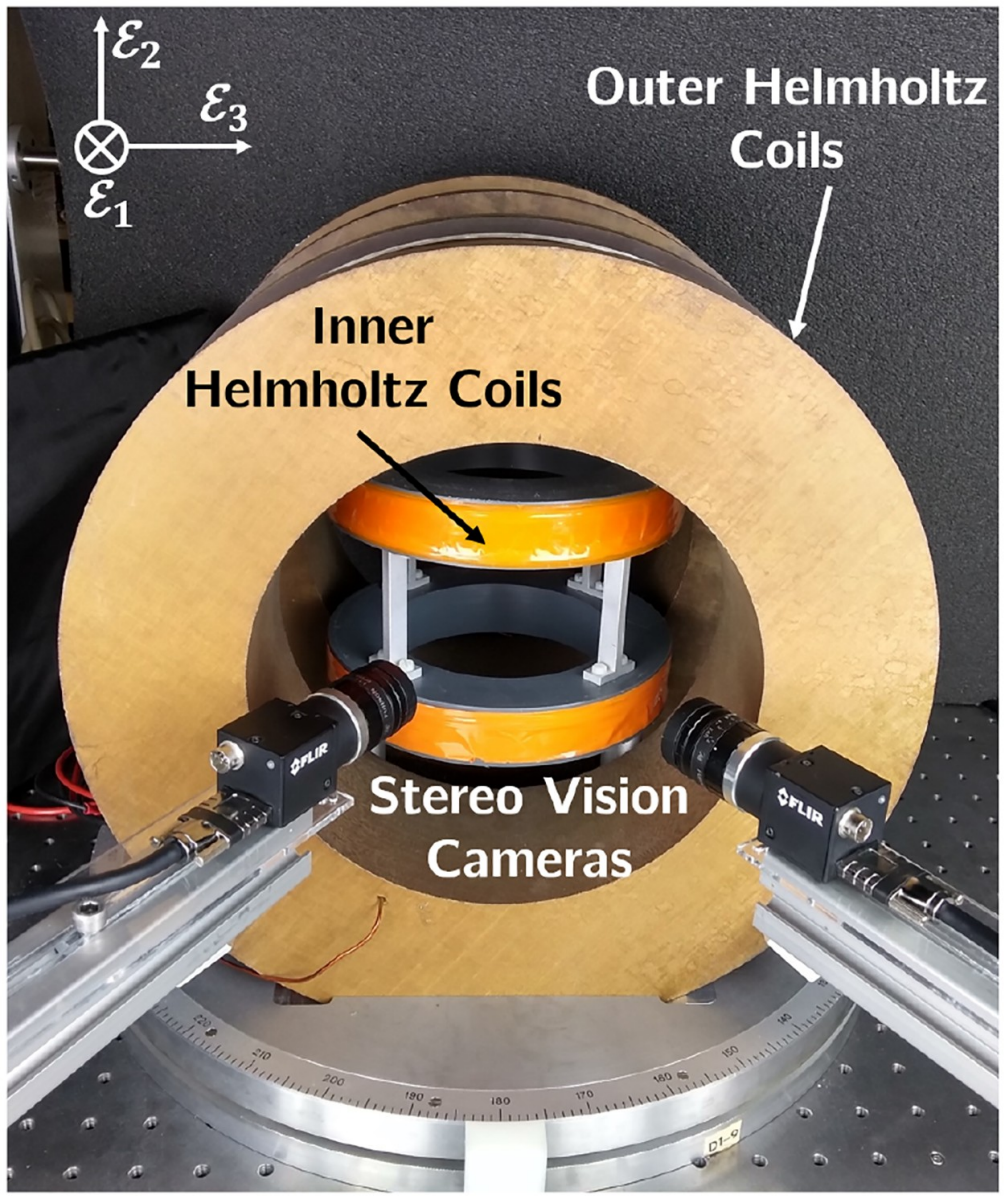
Magnetic field generation setup with the stereo vision cameras. Two nested pairs of Helmholtz coils generate uniform magnetic fields in $E_{1}$ and $E_{2}$-axes direction.

The setup used here consists of two pairs of Helmholtz coils to generate magnetic fields. Each pair consists of two identical electromagnetic coils, as shown in Fig 12. The first pair of coils generates a uniform magnetic field along the $E_{1}$-axis. The second pair of smaller coils are placed inside the first pair to produce a field along the $E_{2}$-axis. Two cameras are placed next to the setup to monitor the side view of the workspace. For image acquisition, we use both cameras in a stereo vision setup to reconstruct 3D views of the manipulator’s motion. The setup produces a maximum magnetic field *B*_*u*_ = 45 mT.

For the first experiment using magnetic actuation, we use the rod with a neodymium magnet with diameter 2 mm, height 4 mm, and magnetisation N45.

First, a magnetic field *B*_*g*_ = 7.75 mT is applied to compensate for the gravity. Then, the tip of the manipulator is induced to rotate in a circle in the $E_{2}E_{3}$-plane using a rotating magnetic field of magnitude *B*_*u*_ = 14.5 mT.

**Fig 13:**
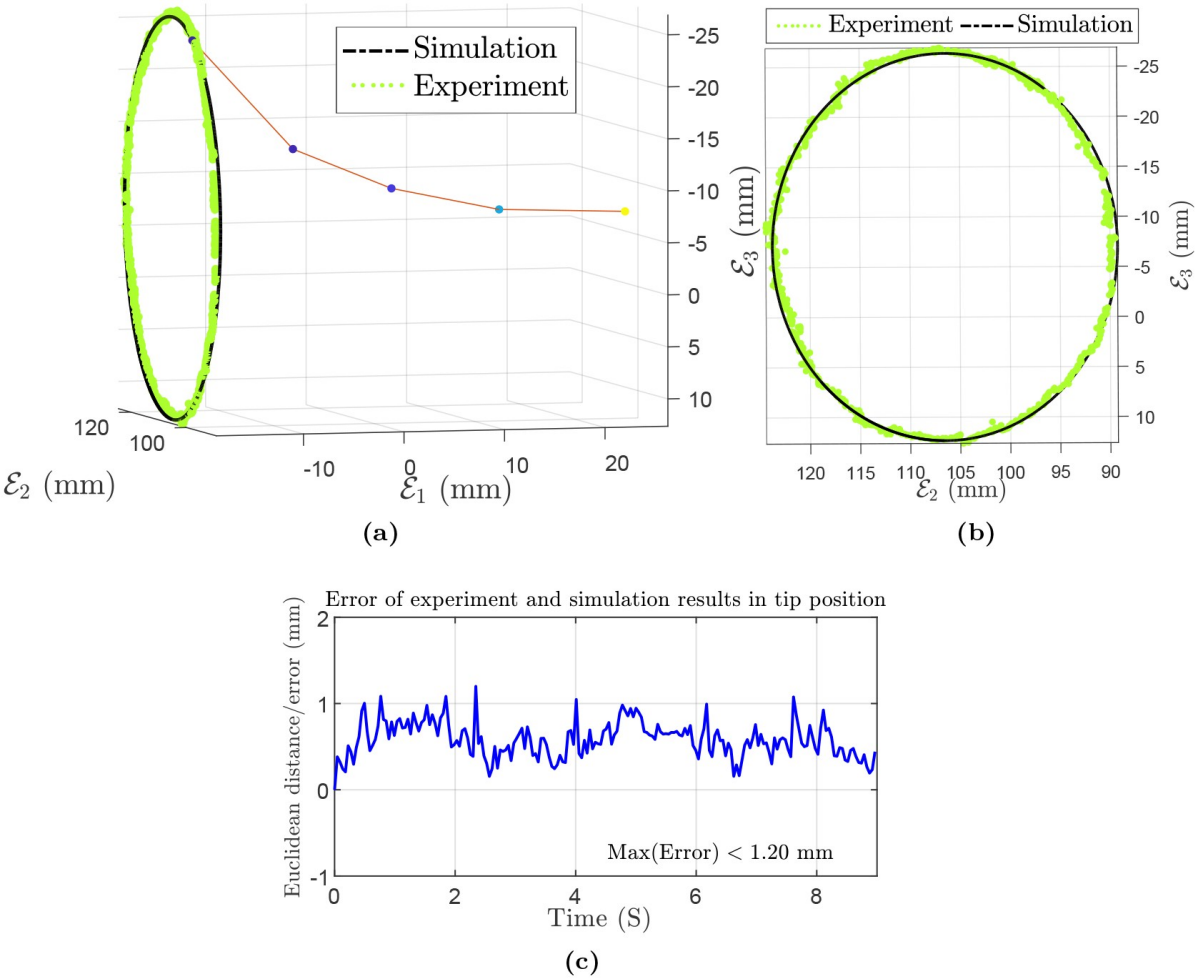
Reconstruction of the scene for circular motion of Polydimethylsiloxane (PDMS) rod with an embedded magnet at the tip: (a,b) 3D experiment and simulation results. (c) Euclidean distance/error of experiment and simulation results in tip position.

The magnetic field produces force and torque *F*_*rot*_ and *τ*_*rot*_, respectively, given by

$$\begin{array}{c} \begin{array}{cc} F_{rot} & =\nabla (m\cdot B_{rot}),\\ \tau_{rot} & =m\times B_{rot} \end{array} \end{array}$$

where *m* is the dipole moment of the tip’s magnet. The dipole moment can be computed as $m=\frac{1}{\mu_{0}}B_{r}V$ in which residual magnetism *B*_*r*_ ∈ [1.32, 1.37] mT, *μ*_0_ is the permeability of vacuum, and the volume of the magnet, *V* = 4*π* mm^3^. Experiment, simulation results and the error are shown in Fig 13. It should be noted that the error plot shows Euclidean norm of the tip position in the experiment and simulation. Also, the maximum and mean absolute errors are 1.20 mm (i.e., 1.43%) and 0.59 mm, respectively. Since we use the same manipulator as the previous experiment, simulation parameters can be found in Table 6.

**Fig 14:**
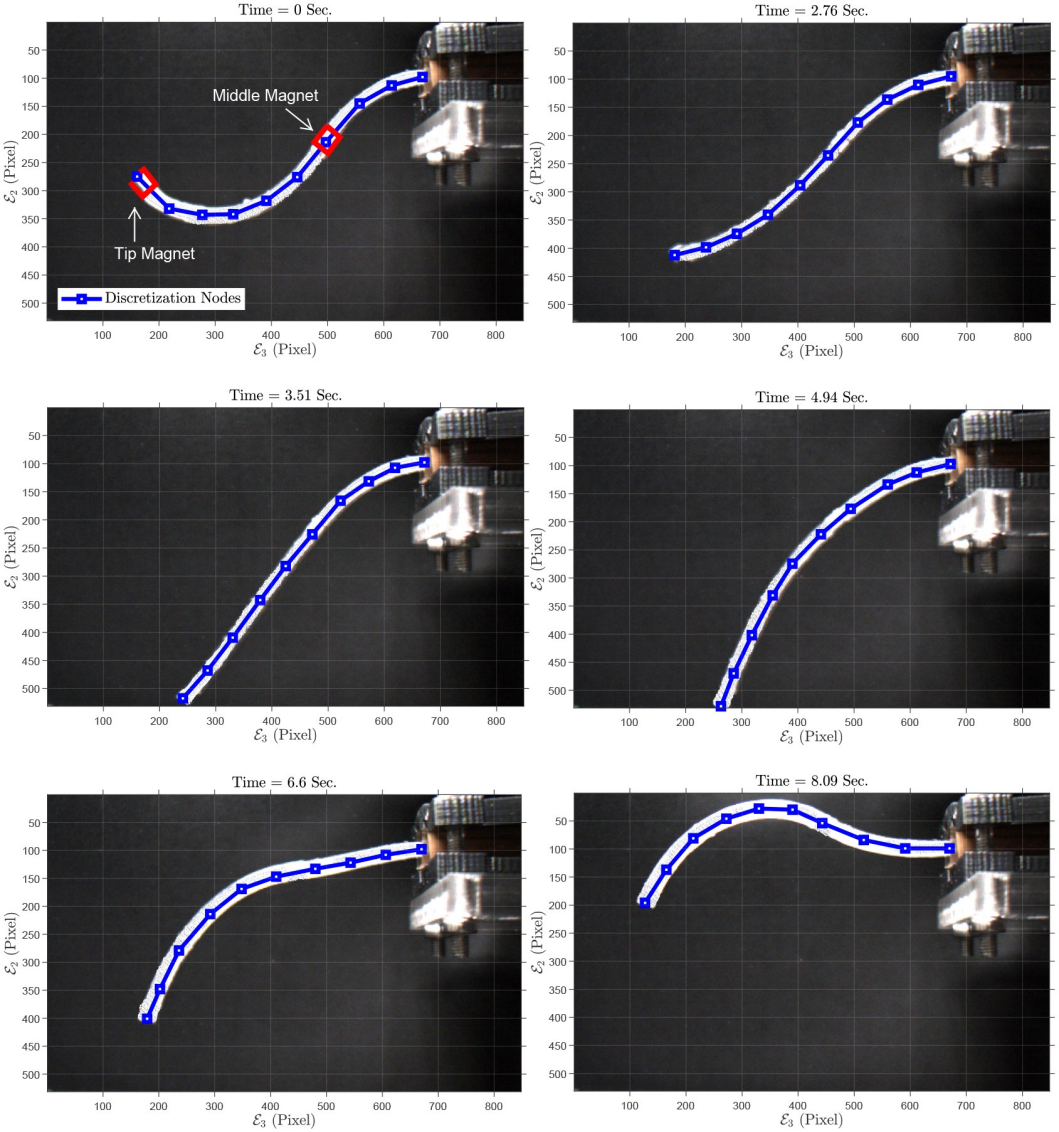
Sample of grabbed images: Polydimethylsiloxane (PDMS) rod with two embedded magnets, 2D experiment: Motion in a plane. Also, 10 discretization points, (blue squares,) are shown on the rod.

As a last experiment, we fabricated a PDMS continuum manipulator with a square cross-section and two embedded permanent magnets, one at the tip and another in the middle—36.1 mm from the tip—of the manipulator. The embedded neodymium magnets are identical cylindrical magnets with different dipole moment’s directions and diameter *D*_*m*_ = 2 mm, height *L*_*m*_ = 3 mm, weight *M*_*m*_ = 7.2 × 10^−5^ kg. The embedded magnets are induced to pursue a prescribed motion in the $E_{2}E_{3}$-plane using a varying magnetic field of initial and final magnitude *B*_*u*_ = 20 mT and 19.85 mT. The initial and some time-evolved configurations of the rod are depicted in Fig 14. It should be noted that analysis of magnetic force and torque follows the same procedure as described above. The specifications of the manipulator are as follows: edge length *a* = 2 mm, length *L* = 85.5 mm. The maximum and mean absolute error for the tip magnet are 1.00 mm (i.e., 2.24%) and 0.15 mm, in $E_{2}$-axis direction, respectively. In $E_{3}$-axis direction, the maximum and mean absolute error for the tip magnet are 1.40 mm (i.e., 5.13%) and 0.33 mm, respectively. The maximum and mean absolute error for the middle magnet are 0.47 mm (i.e., 2.05%) and 0.08 mm, in $E_{2}$-axis direction, respectively. In $E_{3}$-axis direction, the maximum and mean absolute error for the middle magnet are 0.40 mm (i.e., 3.68%) and 0.10 mm, respectively.

**Fig 15:**
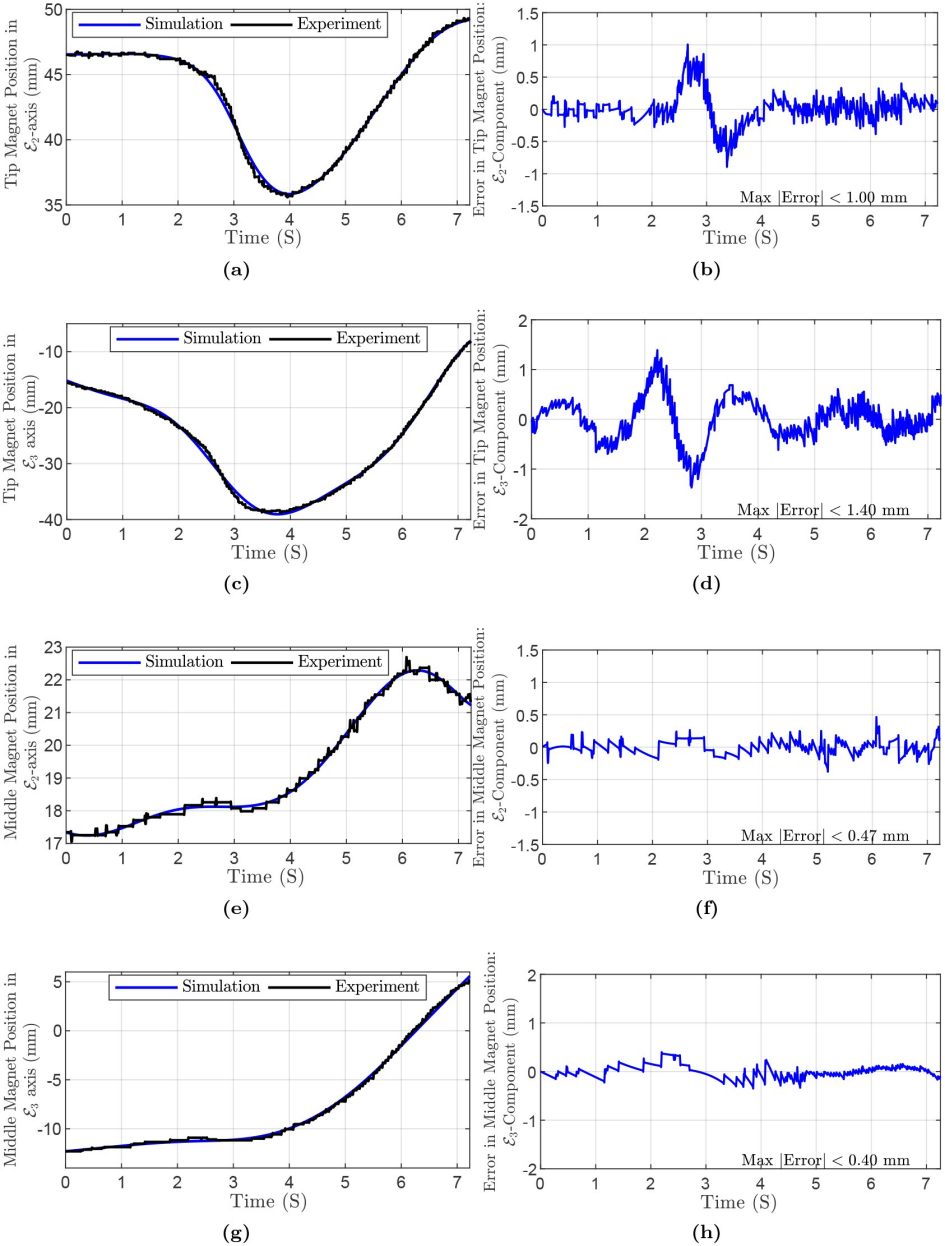
Simulation and experiment results of Polydimethylsiloxane (PDMS) rod with two embedded magnets, 2D experiment: (a) Tip magnet’s position in $E_{2}$-axis direction. (b) Error of tip magnet’s position in $E_{2}$-axis direction. (c) Tip magnet’s position in $E_{3}$-axis direction. (d) Error of tip magnet’s position in $E_{3}$-axis direction. (e) Middle magnet’s position in $E_{2}$-axis direction. (f) Error of middle magnet’s position in $E_{2}$-axis direction. (g) Middle magnet’s position in $E_{3}$-axis direction. (h) Error of middle magnet’s position in $E_{3}$-axis direction.

The position of the tip and middle magnets in the experiment and simulation and also the error is shown in Fig 15. For this experiment, the simulation parameters are summarized in Table 7.

**Table 7:** Simulation parameters in Eqs (10)–(16) and (21) for in-plane experiment of square cross-section PDMS rod (with 2 magnets).

M	$4.40\times 10^{-3}\;\frac{g}{\text{mm}}$
Number of elements	10
$l_{I_{i}}{|}_{i=\{1,2,\cdots ,N\}}$	$\frac{85.5}{10}\;\text{mm}$
*J*_*d*_	diag(0, 1.47, 1.47) g × mm^2^
$E_{3}$	[1, 0, 0]^*T*^
$F_{a_{i}}^{c}{|}_{i=\{1,2,5,\cdots ,N\}}$	${\left[0,0,4.32\right]}^{T}\times 10^{4}\;\frac{\text{g}}{{\text{S}}^{2}}$
$F_{a_{N+1}}^{c}\approx F_{a_{3,4}}^{c}$	${\left[0,0,4.33\right]}^{T}\times 10^{4}\;\frac{\text{g}}{{\text{S}}^{2}}$
*C*_1_	diag(1.46, 0.49, 0.49) × 10^10^
*C*_2_	diag(3.25, 4.87, 4.87) × 10^9^
Time step	1 × 10^−4^
Simulation time	7.5 (S)
R	*I*_3_ × 10^5^
W	*I*_3_ × 10^−1^
Tip and middle magnets weight	0.072 g

## 5 Discussion

We validate our approach by designing and carrying out different experiments with flexible metal rods and polymer-based soft rods. The results are summarized in Table 8.

**Table 8:** Maximum and mean absolute error in the experiments using flexible metal rods (AlSi05) and Polydimethylsiloxane (PDMS) rods.

Experiments		Max. Error	Mean Absolute Error
(1) Flexible rod (AlSi05): in-plane experiment		0.15 mm (i.e., 2.50%)	0.05 mm
(2) Flexible rod (AlSi05): out-of-plane experiment, both axes		0.15 mm (i.e., 2.12%)	0.05 mm
(3) PDMS rod (without magnet): in-plane experiment	$E_{2}$-axis	0.56 mm (i.e., 4.87%)	0.05 mm
	$E_{3}$-axis	0.28 mm (i.e., 4.89%)	0.05 mm
(4) PDMS rod (with magnet): in-plane experiment	$E_{2}$-axis	0.61 mm (i.e., 3.35%)	0.14 mm
	$E_{3}$-axis	0.55 mm (i.e., 1.16%)	0.13 mm
(5) PDMS rod (with magnet): circular motion		1.20 mm (i.e., 1.43%)	0.59 mm
(6) Square cross-section PDMS rod (with 2 magnets): in-plane experiment	Tip magnet: $E_{2}$-axis	1.00 mm (i.e., 2.24%)	0.15 mm
	Tip magnet: $E_{3}$-axis	1.40 mm (i.e., 5.13%)	0.33 mm
	Middle magnet: $E_{2}$-axis	0.47 mm (i.e., 2.05%)	0.08 mm
	Middle magnet: $E_{3}$-axis	0.40 mm (i.e., 3.68%)	0.10 mm

Table 8 demonstrates the maximum and the mean absolute values of the errors. As we observe from this table, the simulation results closely match the experimental responses, i.e., for Experiments 1 and 2 in which the flexible metal rods (AlSi05) are employed, the worst-case errors are < 0.01% of the manipulators’ length. For dynamic Experiments 3 and 4 in which the PDMS rods are used, maximum of errors respectively are 0.95% and 1% of the manipulator’s length. For the polymer rods, higher errors are due to the uncertainties in fabrication and nonlinear elastic properties. In quasi-static Experiments 5 and 6, the manipulators experience large deformations and external loads; the worst-case errors are less than 2% and 1% of the manipulators’ length. It should be pointed out that compared to the manipulators’ length, the mean absolute deviations are small, which reflect the model’s performance.

During the implementation of the modeling approach, it was observed that the number of nodes affects the frequency of motion. Increasing the number of nodes provides a more accurate solution for the frequency of the system. However, the computation time increases significantly with the number of nodes. Therefore, to be able to run the simulations in a reasonable amount of time and with a small number of nodes, frequency shaping was necessary to be able to match the results.

**Fig 16:**
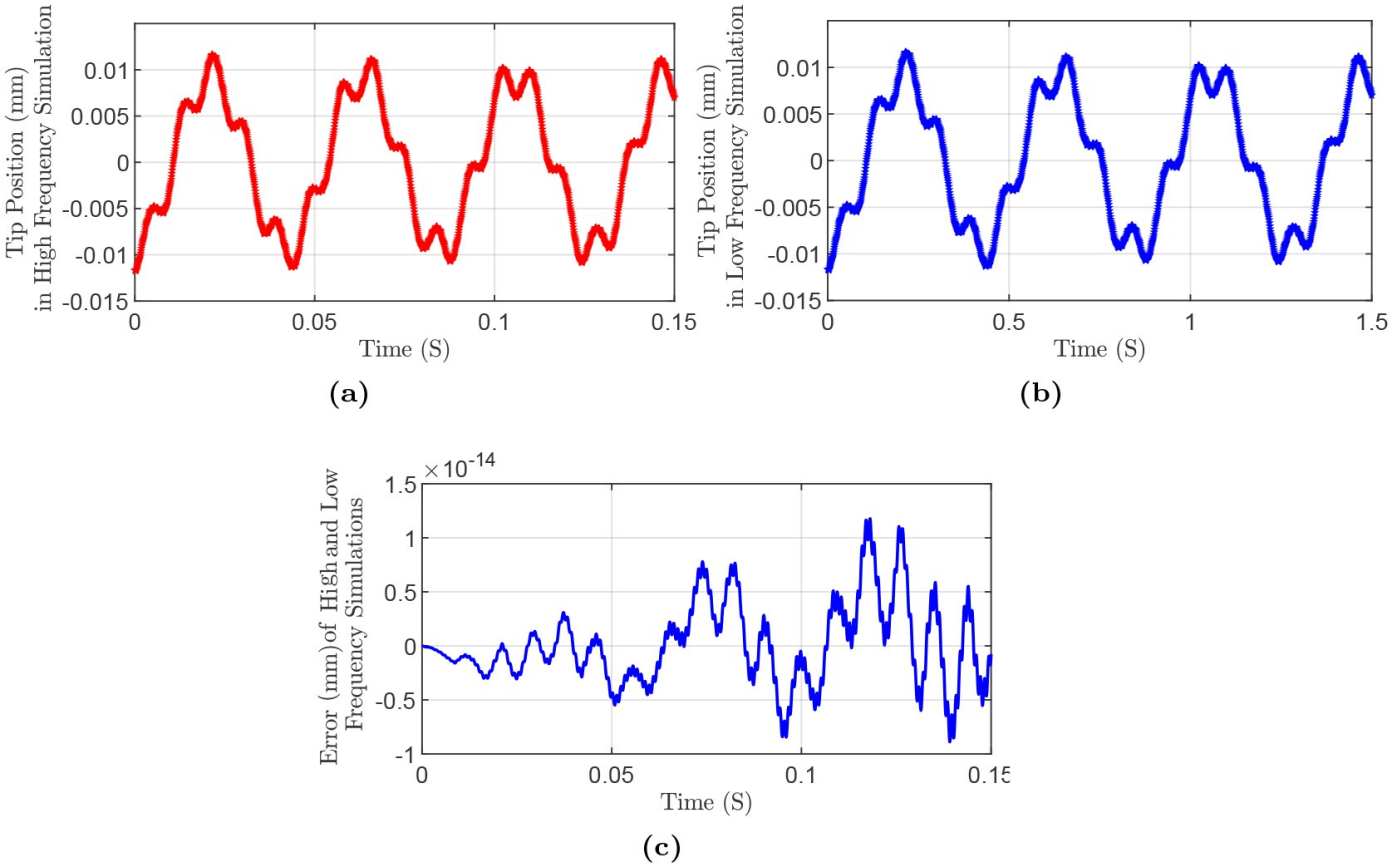
Simulation results for high and low frequency comparison: (a) High frequency. (b) Low frequency. (c) Error of in high and low frequency cases.

The following example shows the motivation behind the frequency shaping of the motion. Consider a manipulator with the following specifications: Length *L* = 0.5 m, mass density $\rho =1000\;\frac{\text{kg}}{m^{3}}$, square cross-section with edge length *a* = 5 cm, Poisson’s ratio *ν* = 0.35, and Young’s modulus *E*_*hf*_ = 5 × 10^4^ KPa in the high frequency case and *E*_*lf*_ = 500 KPa. Fig 16 compares the position between high and low-frequency cases. The base of the manipulator is fixed at the origin. From Fig 16, it is observed that by changing the Young Modulus from *E*_*hf*_ to *E*_*lf*_, tip motion is preserved but in a scaled frequency.

The frequency of the continuum motion is only dependant on parameters such as length, the moment of inertia of the cross-section, Young modulus, and material density. Then, the natural frequency of the continuum manipulator with a fixed end and free tip can be written as

$$\begin{array}{c} \omega_{nf}\propto \sqrt{\frac{EI}{\rho AL^{4}}} \end{array}$$

Consider a manipulator with an equivalent spatial discretization of its central line by $\bar{N}$ elements. ${\omega_{nf}}_{\bar{N}}$ denotes the natural frequency of the manipulator in the simulation with $\bar{N}$ elements, and one has

$$\begin{array}{c} {\omega_{nf}}_{\bar{N}}\propto \sqrt{\frac{\gamma_{corr}EI}{\rho AL^{4}}} \end{array}$$

The correction factor which needs to be multiplied by Young Modulus in the simulation is obtained as

$$\begin{array}{c} \gamma_{corr}=(\frac{{\omega_{nf}}_{\bar{N}}}{\omega_{nf}}{)}^{2} \end{array}$$

By employing this correction factor in the simulation, the effect of the number of discretization elements on the frequency of motion can be eliminated.

High fidelity models are helpful for explaining and predicting the behavior of a system with complex dynamics. However, due to computational constraints, these models may not be employed for closed-loop control purposes in a real-time implementation of robotic applications. Additionally, recent developments in computer simulations demand superior, robust, and efficient numerical frameworks compared to traditional approaches. Discrete geometric mechanics, which are employed in this paper, provides a systematic method to cope with the complexity of continuum manipulators’ dynamics. The necessity of guaranteeing robots’ performance in sensitive applications such as minimally invasive surgeries requires the use of pre-existing knowledge or a model in control architecture to obtain guaranteed and reliable behavior in the presence of disturbances and uncertainties. Although model-free control approaches are easy to implement, they do not provide and ensure any performance level and high control-loop bandwidths.

## 6 Conclusions and future work

This article studies the estimation and model validation problem of continuum manipulators’ dynamics using Lie group variational integrators. Using magnetic actuation, dynamic and static experiments were conducted on manipulators with rigid and soft materials (e.g., Aluminum and PDMS) to illustrate the validity of the presented algorithm for a wide range of experiments.

Due to the lack of knowledge about friction/damping, distributed predictive filters were designed to provide information about the unknown signals. Therefore, the dynamical model equipped with the estimation algorithm is a self-contained generic model for continuum manipulator integration, which provides us with a systematic approach to employ optimal control theory for realistic trajectory planning in the presence of user/environment-specified constraints. The designing of a controller and the parallel variational integration algorithm are to be investigated as future work.

## Supporting information

S1 Appendix Some concepts and definitions on Lie groups and Lie algebra are presented (References [35] and [43]). (PDF)
