# Correction: Dynamic modeling of soft continuum manipulators using lie group variational integration

**DOI:** 10.1371/journal.pone.0242235

**Published:** 2020-11-06

**Authors:** Abbas Tariverdi, Venkatasubramanian Kalpathy Venkiteswaran, Ørjan Grøttem Martinsen, Ole Jacob Elle, Jim Tørresen, Sarthak Misra

The following information is missing from the Funding statement: This research has received funding from the European Research Council (ERC) under the European Union's Horizon 2020 Research and Innovation programme (Grant Agreement #638428—project ROBOTAR). The funders had no role in study design, data collection and analysis, decision to publish, or preparation of the manuscript.
