# Supplementary material for: Dynamic modeling of soft continuum manipulators using lie group variational integration
Source: PLoS One. 2020 Jul 22;15(7):e0236121. doi: 10.1371/journal.pone.0236121 (PMC7375556; doi:10.1371/journal.pone.0236121)
Supplement: S1 Appendix — (PDF) [file pone.0236121.s001.pdf]

Supporting information

S1 Appendix. Some concepts and definitions on Lie groups and Lie algebra are presented (References [35] and [41]).

Preliminaries on Lie groups: A group G is a set of elements with:

- A multiplication, $G \times G \to G$, such that the following properties hold:
    i. The product of g and h is written gh.
    ii. The product is associative, $(gh)k = g(hk)$.
- Identity element e: $eg = g$ and $ge = g$, $\forall g \in G$.
- Inverse operation $G \to G$, so that $gg^{-1} = g^{-1}g = e$.

A Lie group is a smooth manifold G which is also a group and for which the group operations of multiplication, $(g, h) \to gh$ for $g, h \in G$, and inversion, $g \to g - 1$ with $gg^{-1} = g^{-1}g = e$, are smooth functions.

The action of a Lie group G on a manifold M is a group of transformations of M associated to elements of the group G, whose composition acting on M is corresponds to group multiplication in G.

Left and right multiplication by $g \in G$ are denoted by $L_g, R_g : G \to G, L_g(f) = gf, R_g(f) = fg$. The tangent lifted actions $TL_g, TR_g : TG \to TG$ are sometimes denoted as $gv_f := TL_g(v_f)$ and $v_f g := TR_g(v_f)$ for simplicity, where $v_f \in TG$. Similarly, the cotangent lifted actions $T^*L_{g^{-1}}, T^*R_{g^{-1}} : T^*G \to T^*G$ is denoted by $g\alpha_f := T^*L_{g^{-1}}(\alpha_f)$, $\alpha_f g := T^*R_{g^{-1}}(\alpha_f)$, where $\alpha_f \in T^*G$.

Consider $G = SE(3)$, we identify the Lie algebra $\mathfrak{se}(3)$ of G with $\mathbb{R}^3 \times \mathbb{R}^3$ by using the hat map (2). Therefore, the adjoint action is written as:

$$\mathrm{Ad}_{(\mathcal{O}_{a_i}, \mathcal{P}_{a_i})}\Big(\mathcal{O}_{a_k}, \mathcal{P}_{a_k}\Big) = \Big(\mathcal{O}_{a_i}\mathcal{O}_{a_k}, \mathcal{O}_{a_i}\mathcal{P}_{a_k} + \mathcal{P}_{a_i} \times \mathcal{O}_{a_i}\mathcal{O}_{a_k}\Big)$$

also the coadjoint action reads

$$\mathrm{Ad}^*_{(\mathcal{O}_{a_i}, \mathcal{P}_{a_i})^{-1}}\Big(\mathcal{O}_{a_k}, \mathcal{P}_{a_k}\Big) = \Big(\mathcal{O}_{a_i}\mathcal{O}_{a_k} + \mathcal{O}_{P_i} \times \mathcal{O}_{a_i}\mathcal{P}_{a_k}, \mathcal{O}_{a_i}\mathcal{P}_{a_k}\Big)$$

The cotangent lift of left translation $T^*_e L_{(\mathcal{O}_{a_i}, \mathcal{P}_{a_i})} : T_{(\mathcal{O}_{a_i}, \mathcal{P}_{a_i})}G \to T^*_e G$ reads

$$T^*_e L_{(\mathcal{O}_{a_i}, \mathcal{P}_{a_i})}\Big(\mathcal{O}_{a_k}, \mathcal{P}_{a_k}\Big) = \Big(\mathcal{O}^T_{a_i}\mathcal{O}_{a_k}, \mathcal{O}^T_{a_i}\mathcal{P}_{a_k}\Big)$$

where $e = (I, 0)$ is the identity element.
